# Comparative Performance of Wastewater, Clinical, and Digital Surveillance Indicators for COVID-19 Monitoring in Routine Practice: Retrospective Observational Study

**DOI:** 10.2196/70232

**Published:** 2025-11-06

**Authors:** Xinyue Zhang, Zhiqun Lei, Qiuyue Wang, Rui Wang, Jiayao Luo, Everett Jin, Sheng Wei, Qi Wang

**Affiliations:** 1 Department of Epidemiology and Biostatistics, School of Public Health, Tongji Medical College, Huazhong University of Science and Technology, 13 Hangkong Road, Baofeng Street, Qiaokou District, Wuhan, Hubei, 430030, China, 86 27-83692031; 2 St. Mark’s School of Texas, Dallas, TX, United States; 3 School of Public Health and Emergency Management, Southern University of Science and Technology, Shenzhen, Guangdong, China

**Keywords:** surveillance, COVID-19, retrospective study, digital surveillance, wastewater, public health

## Abstract

**Background:**

Public health surveillance systems are critical for decision-making and have been advanced by monitoring infectious diseases.

**Objective:**

This study aims to assess the effectiveness and timeliness of multiple surveillance systems in tracking COVID-19 cases in the postpandemic era.

**Methods:**

Data of COVID-19–reported cases in a southern city of China were collected from the National Notifiable Disease Reporting Information System over a 1-year period, following the easing of the COVID-19 pandemic restrictions (from April 1, 2023, to June 30, 2024) as the operational benchmark. A total of 4 surveillance systems (hospital, wastewater, meteorological, and internet search engine) were integrated into a daily time series. Spearman correlation and 60-day moving window analyses with 7-day lags were used to assess associations. Distributed lag nonlinear models captured nonlinear meteorological effects. Time-series regression models assessed lead effects (0‐7 d) of each surveillance indicator, with and without meteorological adjustment.

**Results:**

Among 4 surveillance systems, 16 variables correlated significantly with reported cases. The nucleic acid amplification test (NAAT) positivity rate showed the strongest correlation, with a coefficient of 0.834 (95% CI 0.803‐0.860). Wastewater surveillance system demonstrated a moderate correlation, with the correlation coefficient of 0.776 (95% CI 0.737‐0.810) for the N gene positivity rate and 0.698 (95% CI 0.648‐0.743) for the N gene concentration. Moving-window analyses confirmed a stable correlation between NAAT positivity and reported cases (median 0.534, IQR 0.394‐0.724; 58% of windows ρ>0.5), while wastewater indicators exhibited greater temporal fluctuation, with the N gene concentration (median 0.585, IQR 0.214‐0.766; 60.8% of windows ρ>0.5) exceeding the N gene positivity rate (median 0.530, IQR 0.222‐0.742; 53.5% of windows ρ>0.5). Time-series analysis identified same-day associations (lag 0) for both NAAT positivity (*β*=.819, 95% CI 0.768‐0.870) and wastewater signals (maximum effect: *β*=1.023, 95% CI 0.931‐1.115). Meteorological factors significantly modified the effect of internet surveillance indicators (*P*<.05), particularly temperature and absolute humidity.

**Conclusions:**

An integrated, multichannel surveillance strategy of leveraging wastewater, clinical, and digital streams with meteorological contextualization can strengthen early warning and situational awareness for respiratory pathogen threats.

## Introduction

Emerging infectious diseases such as COVID-19 have posed significant threats to global health and social security [1,2]. The rapid worldwide transmission of the virus has revealed the fragility of international emergency response mechanisms, underscoring the critical need for establishing effective surveillance systems to swiftly identify, track, and counteract infectious diseases. In the postpandemic era, the challenges of monitoring and managing COVID-19 remain paramount [3]. While traditional surveillance systems are essential, they must be complemented by novel approaches that provide a more nuanced and multifaceted understanding of disease dynamics.

In response to the outbreak of infectious diseases, several surveillance systems developed diverse surveillance strategies to enhance early detection and outbreak control. During the COVID-19 pandemic, wastewater and social media monitoring emerged as powerful tools to complement traditional public health systems [4-6]. Wastewater-based epidemiology, in particular, demonstrated its efficacy in revealing viral circulation trends within communities, often providing early signals of outbreaks before clinical cases are confirmed [7,8]. Meanwhile, social media and internet search data were used to track public interest in disease-related topics, offering insights into potential spatiotemporal patterns of transmission [9-11]. Internet data are also a useful source for syndromic surveillance, and many studies have used it to predict infectious disease trends. For example, a study used Google Ads to target Israeli users searching for COVID-19 symptoms. After clicking the ads, users were connected to a virtual robot that assessed the severity of their symptoms, their demographic information, and their medical history to predict the number of COVID-19 cases and hospitalization rates [12]. Several countries have tended to establish more comprehensive emerging infectious disease surveillance systems from multiple channels. The United States established the National Syndromic Surveillance Program, which leveraged data from sentinel hospitals, community clinics, meteorological sensors, and online media to provide real-time disease surveillance [13]. Similarly, Europe has developed the European Environment and Epidemiology network dedicated to monitoring environmental precursors to epidemics, along with the Threat Tracking Tool for continuous online media and disease bulletin surveillance [14-16]. These systems have highlighted the importance of combining traditional public health infrastructure with novel technological approaches to anticipate and mitigate future epidemics.

Following the conclusion of the public health emergency, the vast majority of COVID-19 monitoring data sources are expected to remain accessible. In 2023, as part of the transition of COVID-19 from emergency responses to routine public health undertakings, the United States Centers for Disease Control and Prevention (CDC) established the Coronavirus and Other Respiratory Viruses Division [17]. Despite the establishment of diversified surveillance systems in many countries, the comparison between different systems needs further study. It has remained unclear which of these systems provides timely and dependable information on the evolution of infectious diseases endemic. This knowledge gap hinders the ability to optimize public health responses and allocate resources efficiently [18]. The ongoing surveillance of COVID-19 has remained a key focus in the postpandemic era. By identifying the strengths and weaknesses of various surveillance systems, policymakers and health care professionals can make informed decisions regarding the implementation and refinement of disease monitoring strategies [19]. Therefore, a comprehensive assessment of these surveillance systems’ performance and their role in tracking the COVID-19 pandemic is of great significance.

After the policy shift of COVID-19 on January 6, 2023, in China, it has presented a unique opportunity to study the correlation between the endemic evolution and the effectiveness of various surveillance systems in a real-world setting. In this study, we analyzed the correlations between the COVID-19 endemics and various surveillance systems in a Chinese southern city following this transition. Finally, we evaluated the timeliness, stability, and lead-lag relationships of different surveillance systems to clarify their distinct roles and contributions in monitoring the prevalence of infectious diseases. Our findings aim to inform the design of an effective multichannel surveillance framework for the postpandemic era.

## Methods

### Study Design

We first described the daily cases reported 1 year following the transition from emergency response to long-term COVID-19 disease management. The dataset was used as the benchmark for correlation analysis with the results from 4 surveillance systems, including a hospital surveillance system, a wastewater surveillance system, a meteorological surveillance system, and an internet search engine system (Table S1 in Multimedia Appendix 1).

We integrated traditional public health surveillance data (eg, laboratory confirmations and clinical reports) with novel digital proxies and contextual environmental data. While clinical reports and wastewater monitoring were direct components of public health surveillance systems, internet search metrics, such as Baidu Search Index (BSI), were used as digital syndromic surveillance tools that proxy population-level health-seeking behavior. Meteorological factors were included as contextual confounding variables to control for their potential influence on both virus transmission and reporting behavior.

### COVID-19–Reported Cases

Data were initially sourced from the National Notifiable Disease Reporting Information System (NDRIS). Following the 2003 SARS outbreak, China established the web-based NDRIS for quick case identification and response [20]. Since 2008, all notifiable infectious disease data (including COVID-19 since January 2020) have been electronically reported to the China CDC within 24 hours. The system covered all 31 mainland provinces, with individual case data standardized by the National Health Commission’s guidelines. We analyzed COVID-19 confirmed cases reported by medical institutions via the NDRIS from April 1, 2023, to June 30, 2024. All individual case details were de-identified.

### Surveillance Systems

#### Hospital Surveillance System

Data from the hospital surveillance system were derived from 2 key sources. One was the daily number of patient visits to fever clinics across all major health care institutions in the city. The other was the positivity rate of the SARS-CoV-2 nucleic acid amplification test (NAAT) from sentinel surveillance. The sentinel network comprised 2 national-level and 13 provincial-level influenza surveillance hospitals, which provided a robust platform for monitoring COVID-19 among patients with influenza-like symptoms, ensuring good population coverage. The surveillance system was separate from, but complementary to, the NDRIS. The former was designed for situation awareness, while the latter was used for mandatory case reporting. We intentionally focused on outpatient syndromic metrics (fever clinic visits and NAAT positivity rate) as leading indicators of community transmission. Hospital admission data were excluded as they represented a lagging indicator of severe outcomes and fell outside this study’s primary scope of evaluating endemic surveillance systems.

#### Wastewater Surveillance System

The wastewater sampling data for COVID-19 surveillance, which included the total detection rate and the counts of SARS-CoV-2 viral genome copies (copies/mL) in wastewater samples from sewage treatment plants, were obtained from statistics provided by the city’s CDC.

#### Meteorological Surveillance System

Meteorological surveillance data were collected from the China Meteorological Data Sharing Service Center [21]. We cleaned the meteorological data from the city from March 1, 2023, to June 30, 2024. The data comprised the mean temperature per day (Tmean), mean relative humidity per day (RHmean), mean air pressure per day (Pmean), mean wind speed per day (WSmean), mean precipitation per day (PRCPmean), and mean visibility range per day (VISmean). The mean absolute humidity (AHmean) was calculated based on the Tmean and RHmean, and the formula was as follows [22]:


$$\begin{array}{c} AHmean=\left\{6.112\times exp\left[\left(17.67\times Tmean\right)/\left(Tmean\pm 243.5\right)\right]\times \;RHmean\times 2.1674\right\}/\left(273.15+Tmean\right) \end{array}$$

#### Internet Search Engine System

The Baidu search engine has been one of the most popular search engines in China, and BSI reflects the keyword search patterns of numerous Baidu users. In this study, we selected 10 keywords related to COVID-19, with 5 being symptom-related and 5 being name-related [23]. The timeframe for data collection was set to align with the period covered by the data from the case reporting surveillance system. To capture the search behavior of internet users, we retrieved BSI data encompassing searches conducted on personal computers and personal mobile phones. A detailed explanation of the process used to gather BSI data was shown in Multimedia Appendix 2.

### Monitoring Period

Our analysis focused on the comparison between April 1, 2023, and June 30, 2024. This timeframe centered on China’s policy shift in COVID-19 management, transitioning toward routine prevention and control measures. By the end of the study period, SARS-CoV-2 showed patterns consistent with endemic circulation in the study region. Nevertheless, episodic local epidemic waves continued to occur and formed the focus of our analysis. The surveillance systems we selected for this study have implemented standardized data collection and reporting protocols. The discrepancy in the time frames arose because the meteorological surveillance system covered a slightly extended period (March 1, 2023, to June 30, 2024) to ensure comprehensive monitoring and to capture potential early signals of disease activity. All time series were aligned by calendar date. We assessed data availability for each surveillance stream across the entire study period and report exact sample sizes and completeness in Table S2 in Multimedia Appendix 1 (data availability and completeness). Reported cases had 48 of 60 (10.50%) days of missing data, and missing values were imputed using linear interpolation prior to analyses.

### Statistical Analysis

#### Correlation Analysis

The Spearman correlation test was used between daily reported case counts and multiple surveillance systems throughout the entire monitoring period, as well as on a moving basis for 60-day periods. This nonparametric approach was chosen for its suitability to our dataset, which encompassed nonnormally distributed variables and potential outliers. Unlike methods that assume strictly linear relationships, the Spearman coefficient assesses the strength and direction of monotonic associations, thereby providing a more robust and reliable measure of correlation in the context of our epidemiological analysis. A correlation coefficient close to +1 or –1 indicates a strong correlation, while a coefficient close to 0 indicates a weak correlation. This also provided a robust foundation for selecting variables for subsequent detailed analysis using a distributed lag non-linear model (DLNM).

Correlation analysis over a 60-day span was used to assess variations in time-series correlations beyond short durations and the entire monitoring period. While the reporting system is considered relatively stable, multiple factors could influence case ascertainment over time. For instance, a decrease in the severity of disease following vaccination might diminish the incentive for seeking NAAT, or the prevalence of influenza, which presents with symptoms similar to COVID-19, and during the flu season could impact the seeking of medical attention and subsequent reporting of the disease. The distribution of correlation coefficients across these rolling windows was summarized using the median and IQR, and, additionally, the proportion (%) of 60-day windows was calculated, in which ρ exceeded 0.5. This approach was chosen because the median provides a robust measure of the central tendency of the correlations that is resistant to potential outliers in individual 60-day periods, and the IQR effectively captures the middle 50% of the variability in the strength of the association over time. The proportion of windows with ρ>0.5 provided an interpretable measure of temporal consistency (stability) of the association. In addition, correlation analysis was repeated throughout the entire cycle with a lag from –7 to +7 days, retaining the values of reported cases on original dates while shifting comparative variables. Specifically, a negative lag (eg, –*k* days) evaluates the predictive capacity of a surveillance system by correlating its measurement on day *t* with cases reported on a future day *t*+*k*. A significant correlation at a negative lag suggested that the surveillance signal leads the official case reporting, potentially serving as an early warning indicator. Conversely, a positive lag (eg, +*k* days) evaluates a retrospective association by correlating a surveillance measurement from a past day *t*–*k* with cases reported on day *t*.

#### Time-Series Model

DLNM was used to account for the nonlinear and delayed effects of meteorological factors [24]. Meteorological variables, including Tmean, RHmean, AHmean, Pmean, WSmean, PRCPmean, and VISmean, were incorporated using a second-degree polynomial basis for the exposure-response dimension. The lag structure was modeled with a natural cubic spline with 2 internal knots, capturing effects up to 7 days. Long-term trends and seasonality were adjusted for using a natural spline of time with 4 degrees of freedom. Day of the week and holiday effects were included as categorical factors.

The quasi-Poisson distribution was used to accommodate overdispersion in daily case counts. All surveillance indicators and meteorological covariates were standardized to z-scores (mean 0, SD 1) prior to inclusion in the models to facilitate interpretation of effect sizes. No offset term was included as the outcome was daily case counts, and the study population remained constant throughout the observation period. The model specification was as follows:


$$\log\left[E\left(Y_{t}\right)\right]=\beta_{0}+\sum \left[s\left(x_{i},lag\right)\right]+ns\left(time,df=4\right)+year+DOW+holiday$$

where *Y*_*t*_ represented the daily case count at time *t*, *β*_*0*_ was the intercept, *s* (*x*_*i*_,*lag*) represented the cross-basis function for each exposure variable *x*_*i*_ (including both meteorological and surveillance variables) incorporating both exposure-response and lag-response dimensions, *ns* (*time*, *df*=4) was the natural spline of time with 4 degrees of freedom per year to control for long-term trends and seasonality, *DOW* represents day-of-week effects, and *holiday* represented holiday effects.

Given the exploratory nature of this multichannel surveillance comparison and the numerous correlations and regression coefficients evaluated, *P* values should be interpreted with caution as indicators of association strength rather than definitive proof. We applied false discovery rate correction to account for multiple testing while maintaining reasonable statistical power for detecting meaningful associations in this hypothesis-generating context.

The lead effects of multiple surveillance variables were evaluated by incorporating lagged terms of each surveillance variable into the time-series model. We assessed their predictive capabilities from 0 to 7 days ahead of official case notifications. The analysis systematically accounted for potential confounding by meteorological factors, enabling a clearer understanding of the intrinsic association between surveillance signals and disease incidence. The results were presented graphically in timeline plots, which showed the estimated effect sizes and 95% CIs for each lead period, both with and without adjustment for meteorological confounders. Positive coefficients signify that an increase in the surveillance signal correlates with a subsequent rise in case reports, after accounting for seasonal trends, day of the week, and holiday effects. The adjustment ensured a direct comparison of the predictive use of each surveillance system over time and highlighted the importance of adjusting for meteorological variables to obtain unbiased effect estimates.

Potential effect modification by meteorological variables was assessed by introducing interaction terms between surveillance indicators and meteorological factors into adjusted models. Statistically significant interactions were further visualized to illustrate how the relationship between surveillance signals and case counts varied under different meteorological conditions.

All time-series data were smoothed. Correlation analyses in this study were performed using Python software (version 3.12.0; Python Software Foundation). The time-series model was performed using R Studio and R software (version 4.3.0; R Core Team), specifically the R package “dlnm”. *P*<.05 was considered statistically significant.

## Results

### Description of Data From all Surveillance Systems

From April 1, 2023, to June 30, 2024, a cumulative total of 62,236 cases were reported, predominantly characterized by sporadic occurrences. The first peak occurred in 2023 from late April to the end of June. Since the beginning of 2024, the number of reported cases has consistently remained low. February 18, 2024, the first working day after the Spring Festival holiday, marked the onset of an upward trend in reported cases. The second peak was reached between March and June, followed by a fluctuating downward trend, as illustrated in Multimedia Appendix 3.

Starting from January 8, 2023, the daily patient volume at fever clinics in medical institutions began to fluctuate and decrease, exhibiting a pattern of varying changes. It reached 3 peaks in March, May, and December of 2023. Since November 20, 2023, the patient volume had rapidly increased, reaching its peak on December 5, 2023, and remaining high for 52 days. The characteristics of low-level fluctuations were observed since February 2024. Peaks in the positivity rate of NAAT occurred from late April to late June and from July to mid-October in 2023. In January 2024, the positivity rate dropped to around 2% and showed a fluctuating upward trend, reaching its peak in late March (Multimedia Appendix 3). Wastewater SARS-CoV-2 detection rates declined early in 2023 before rising significantly post April 20, 2023, remaining elevated until June 2023 before trending downward with fluctuations. In 2024, from May 27 to June 3, the positive detection rate of sewage experienced a significant fluctuation and increase, with a weekly average increase of more than 30%, reaching the alert level. From June 6 to June 10, the overall positive detection rate fluctuated at a high level. From June 13 to June 24, it showed a trend of fluctuating decrease. The virus concentration followed a similar pattern (Multimedia Appendix 3). Daily weather variables and COVID-19–related BSI trends were detailed in Multimedia Appendix 3.

### Correlation of Surveillance Systems

Table 1 shows the correlations between all surveillance datasets and the reported cases throughout the entire monitoring period (from April 1, 2023, to June 30, 2024). Most of the correlations were significant (*P*<.05), indicating evidence of relationships. Among them, the positive rate of NAAT showed a strong correlation (ρ=0.834, 95% CI 0.803‐0.860). The 4 variables of the wastewater surveillance system also exhibited a moderate correlation (all ρ>0.5; all *P*<.001). The correlation results of meteorological surveillance data revealed a moderate positive correlation between ambient temperature (ρ=0.346, 95% CI 0.263‐0.425), absolute humidity (ρ=0.330, 95 %CI 0.245‐0.409), and COVID-19–reported cases; a low positive correlation was observed between visibility and COVID-19–reported cases (ρ=0.179, 95% CI 0.089‐0.226). Atmospheric pressure exhibited a moderate negative correlation (ρ=−0.322, 95% CI –0.402 to –0.237).

**Table 1.** Spearman correlation analysis between different surveillance systems between April 2023 and June 2024.

Surveillance systems and datasets	Coefficient (95% CI)	*P* value
Operational benchmark		
Cases reported	1 (1 to 1)	—^a^
Comparator surveillance systems		
Hospital surveillance system		
Visits	–0.088 (–0.179 to 0.003)	.06
Positive rate	0.834 (0.803 to 0.860)	<.001
Wastewater surveillance system		
N gene concentration	0.698 (0.648 to 0.743)	<.001
N gene positive rate	0.776 (0.737 to 0.810)	<.001
ORF1ab gene concentration	0.574 (0.509 to 0.632)	<.001
ORF1ab gene positive rate	0.661 (0.606 to 0.710)	<.001
Meteorological surveillance system		
Tmean^b^	0.346 (0.263 to 0.425)	<.001
Pmean^c^	–0.322 (–0.402 to –0.237)	<.001
RHmean^d^	0.001 (–0.091 to 0.093)	.98
AHmean^e^	0.330 (0.245 to 0.409)	<.001
WSmean^f^	0.062 (–0.030 to 0.152)	.19
VISmean^g^	0.179 (0.089 to 0.266)	<.001
PRCPmean^h^	0.048 (–0.044 to 0.139)	.31
Internet Search Engine System		
BSI^i^1, Baidu search rank for “fever”	0.077 (–0.015 to 0.167)	.10
BSI2, Baidu search rank for “cough”	–0.186 (–0.274 to –0.096)	<.001
BSI3, Baidu search rank for “sore throat”	–0.047 (–0.138 to 0.045)	.32
BSI4, Baidu search rank for “weakness”	0.154 (0.064 to 0.243)	<.001
BSI5, Baidu search rank for “diarrhea”	0.197 (0.108 to 0.284)	<.001
BSI6, Baidu search rank for “COVID-19”	0.290 (0.204 to 0.372)	<.001
BSI7, Baidu search rank for “novel coronavirus”	0.244 (0.156 to 0.328)	<.001
BSI8, Baidu search rank for “COVID-19 pneumonia”	0.164 (0.074 to 0.252)	<.001
BSI9, Baidu search rank for “novel coronavirus pneumonia”	0.061 (–0.031 to 0.152)	.19
BSI10, Baidu search rank for “Omicron”	0.137 (0.046 to 0.226)	.003

a Not applicable.

b Tmean: mean temperature per day.

c Pmean: mean air pressure per day.

d RHmean: mean relative humidity per day.

e AHmean: mean absolute humidity per day.

f WSmean: mean wind speed per day.

g VISmean: mean visibility per day.

h PRCPmean: mean precipitation per day.

i BSI: Baidu Search Index.

**Figure 1.**
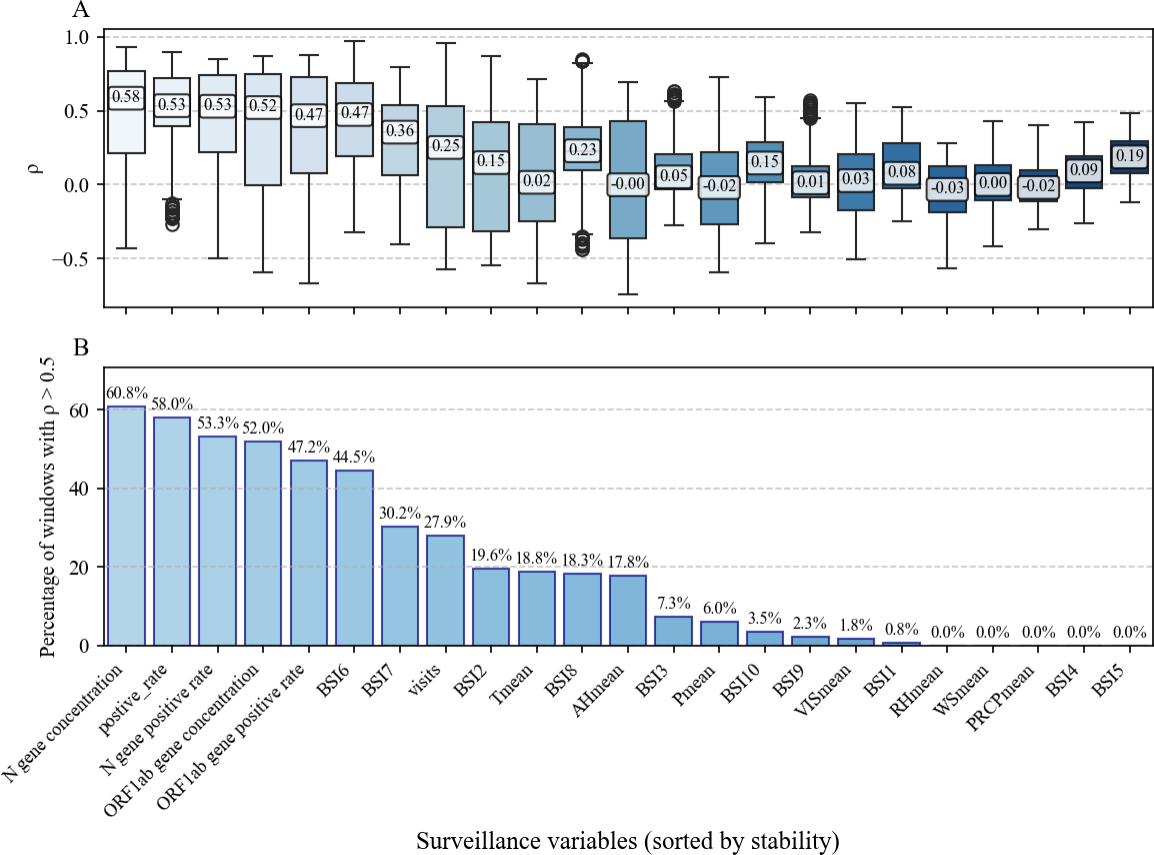
Correlation analyses of different variables with a moving 60-day window. (A) Distribution of rolling-window ρ (boxplots; median, IQR). (B) Proportion of 60-day windows with ρ>0.5 (stability measure). AHmean: mean absolute humidity per day; BSI: Baidu Search Index; Pmean: mean air pressure per day; PRCPmean: mean precipitation per day; RHmean: mean relative humidity per day; Tmean: mean temperature per day; VISmean: mean visibility per day; WSmean: mean wind speed per day.

To evaluate time-series correlation with 60-day moving windows, median Spearman values and the IQR of Spearman estimates were used for comparison with the reported cases (Figure 1). The wastewater data showed a positive correlation with reported cases, but with significant variability. Among the sewage monitoring indicators, N gene concentration was the most consistently and strongly correlated indicator, with 60.8% of rolling windows showing ρ>0.5 (median 0.585, IQR 0.214‐0.766) and the N gene positivity rate in 53.3% of rolling windows (median 0.530, IQR 0.222‐0.742). *ORF1ab* gene measures were slightly weaker but still relatively stable (*ORF1ab* gene concentration: 52%; *ORF1ab* gene positive rate: 47.2%). Among the hospital surveillance variables, the daily NAAT positive rate (positive_rate) was consistently strongly correlated indicator, with 58% of rolling windows showing ρ>0.5 (median 0.534, IQR 0.394‐0.724). The correlation between the number of fever clinic consultations and reported cases showed substantial temporal variability (27.9% of ρ>0.5 of rolling windows). The correlation approached zero around September 2023 and moderate negativity from October 2023 to March 2024. The median correlation coefficients between meteorological surveillance data and reported cases were mostly close to 0. Several BSIs showed meaningful associations: BSI6 (44.5%) and BSI7 (30.2%) had the highest shares among the BSI series, while other BSI terms declined in stability (BSI2 19.6%, BSI8 18.3%, BSI3 7.3%, BSI10 3.5%, BSI9 2.3%, BSI1 0.8%, BSI4 and BSI5 0%). The keyword-based BSI for name-related queries (eg, “COVID-19”) demonstrated stronger and more consistent correlations than symptom-related queries (median 0.475, IQR 0.192‐0.685). Median correlation coefficients for meteorological variables were generally close to zero, and their stability was low: temperature (Tmean) 18.8% of ρ>0.5 of rolling windows, absolute humidity (AHmean) 17.8%, visibility (VISmean) 1.8%, wind speed (WSmean), and precipitation (PRCPmean) 0.0%. These findings indicated the limited and inconsistent predictive value of meteorological variables when considered alone (Multimedia Appendix 4).

**Figure 2.**
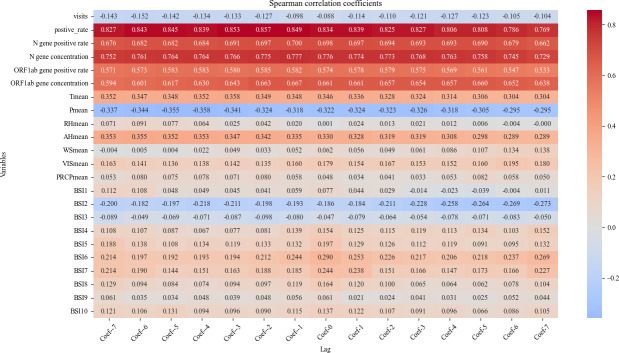
Correlation analyses of different variables with a 7-day lag before and after. Negative lag values denote surveillance data leading reported cases (predictive effect), while positive lag values denote surveillance data lagging behind reported cases. AHmean: mean relative humidity per day; BSI: Baidu Search Index; Pmean: mean air pressure per day; PRCPmean: mean precipitation per day; RHmean: mean relative humidity per day; Tmean: mean temperature per day; VISmean: mean visibility per day; WSmean: mean wind speed per day;

Correlation analyses showed the same association results with the lag (–7 to +7 d). Notably, the weather variables from the meteorological surveillance system demonstrated a stronger correlation coefficient with a longer lag period, indicating a stronger correlation with the reported cases, as detailed in Figure 2 and Multimedia Appendix 4.

### Temporal Lead Effects of Surveillance Indicators

To evaluate the predictive use of the selected surveillance indicators, we analyzed their association with reported case counts across a range of lead times from 0 to 7 days. The results of time-series regression analyses are summarized below.

**Figure 3.**
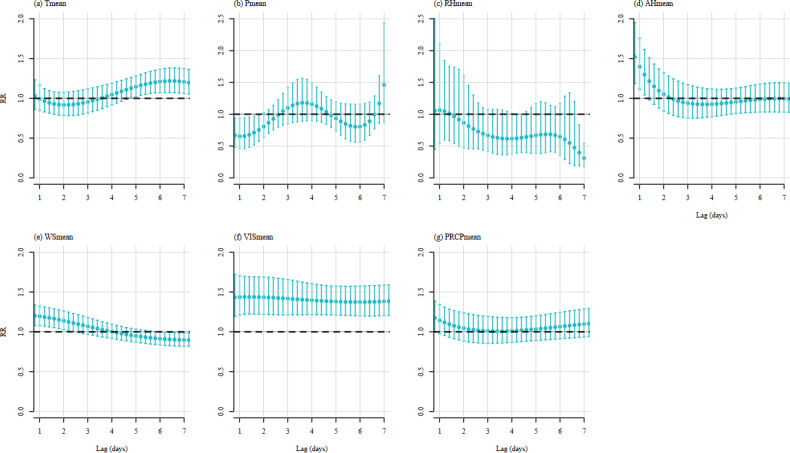
Distributed lag nonlinear models of meteorological factors with a 7-day lag before. (**A**) Tmean, (**B**) Pmean, (**C**) RHmean, (**D**) AHmean, (**E**) WSmean, (**F**) VISmean, and (**G**) PRCPmean. Vertical bars indicate 95% CIs. AHmean: mean relative humidity per day; Pmean: mean air pressure per day; PRCPmean: mean precipitation per day; RHmean: mean relative humidity per day; Tmean: mean temperature per day; VISmean: mean visibility per day; WSmean: mean wind speed per day.

DLNM analysis with a 7-day lag was to explore the association between each meteorological factor and the reported cases, and the results were consistent with the correlation analyses, as detailed in Figure 3.

**Figure 4.**
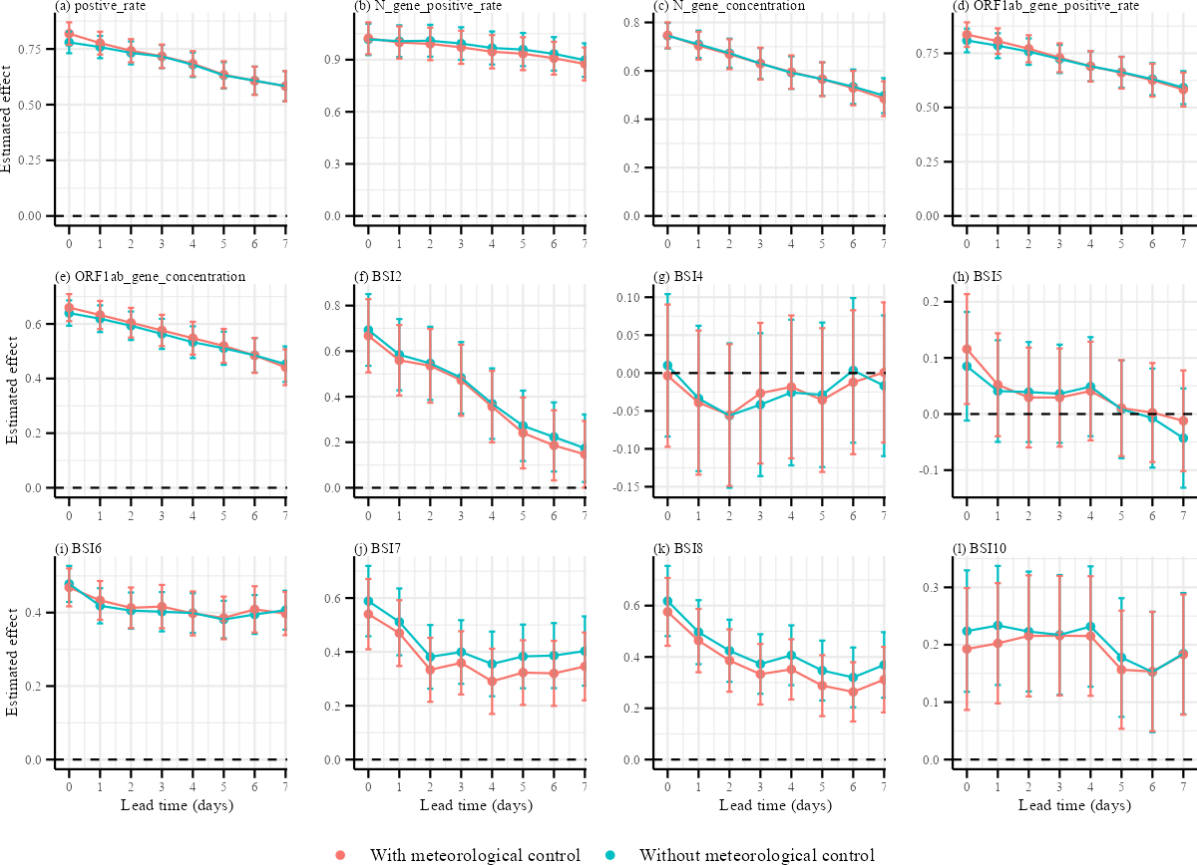
Estimated effects of multiple surveillance indicators on reported COVID-19 cases across lead times, with and without meteorological adjustment. Twelve panels (a–l) depict effect estimates (β coefficients) from time-series regression models for different lead times (0‐7 d), including (**A**) nucleic acid amplification test (NAAT) positivity rate, (**B**) N gene positivity rate, (**C**) N gene concentration, (**D**) *ORF1ab* gene positivity rate, and (**E**) *ORF1ab* gene concentration. Panels (F–L) depict Baidu Search Index (BSI) variables: (**F**) BSI2, (**G**) BSI4, (**H**) BSI5, (**I**) BSI6, (**J**) BSI7, (**K**) BSI8, and (**L**) BSI10. Orange lines represent models adjusted for meteorological factors (temperature and absolute humidity), while teal lines represent unadjusted models. Vertical bars indicate 95% CIs. Effects represent the change in daily case counts per SD value increase in each surveillance variable. BSI: Baidu Search Index.

The timeline plots revealed key insights into the estimated effect sizes for each standardized variable derived from quasi-Poisson regression models (Figure 4). Most surveillance variables demonstrated their strongest associations at a lead time of 0 days, indicating a high degree of contemporaneous alignment with case reporting. The analysis identified N gene positive rate of wastewater surveillance as the most consistent leading indicator (maximum effect size: *β* = 1.023, 95% CI 0.931‐1.115 at lag 0 d), followed by the positivity rate of NAAT (maximum effect size: *β* = .819, 95% CI 0.768‐0.870 at lag 0 d), supporting its potential use in contemporaneous alignment with short lead potential. Importantly, comparisons of effect estimates before and after adjusting for meteorological confounders (eg, Tmean and AHmean) showed that relationships for certain variables, particularly internet search indicators, were significant. This highlighted the critical role of controlling for meteorological confounding in deriving unbiased effect estimates, as detailed in Multimedia Appendix 5.

**Figure 5.**
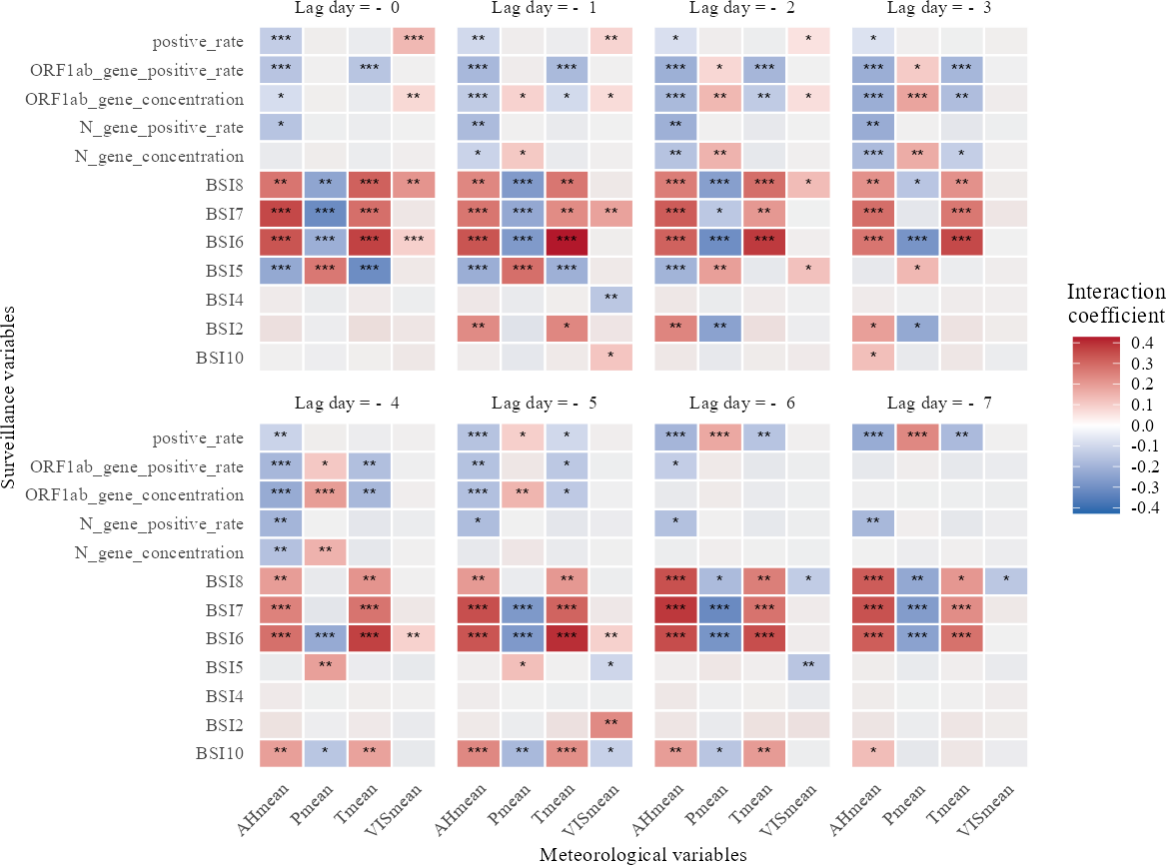
Heatmap of interaction coefficients between meteorological variables and surveillance indicators across different lead days. Each cell shows the interaction coefficient quantifying how meteorological conditions modify the association between a given surveillance indicator (rows) and reported COVID-19 cases at specific lead days (columns: lag 0 to lag −7). Color scale indicates coefficient magnitude and direction (red=positive interaction; blue=negative interaction); intensity reflects effect size. Light gray cells denote statistically nonsignificant interactions (*P*≥.05). Asterisks denote statistical significance (* *P*<.05; ** *P*<.01; *** *P*<.001). AHmean: mean relative humidity per day; BSI: Baidu Search Index; Pmean: mean air pressure per day; PRCPmean: mean precipitation per day; RHmean: mean relative humidity per day; Tmean: mean temperature per day; VISmean: mean visibility per day; WSmean: mean wind speed per day.

Figure 5 summarizes the interaction coefficients between meteorological factors and multiple surveillance indicators over lead times of 0 to −7 days. Overall, Tmean and AHmean exhibited the most frequent and pronounced modifications of surveillance signals, whereas Pmean and VISmean showed weaker and less consistent interactions. Interaction effects were temporally heterogeneous: many significant interactions clustered at short negative lags (typically lag −1 to lag −3), indicating that recent meteorological conditions can modify surveillance-case associations within a few days. Laboratory- and wastewater-based indicators (NAAT positive rate, N gene and *ORF1ab* gene positivity, and concentration) displayed relatively few significant interactions overall, suggesting that these signals were robust to short-term meteorological variation. By contrast, internet-search indicators (BSIs) showed more numerous and larger interaction coefficients, with several positive interactions with Tmean and negative interactions with AHmean reaching statistical significance (*P*<.05), as detailed in Multimedia Appendix 5.

## Discussion

### Principal Findings

In this city-level study spanning April 1, 2023, to June 30, 2024, we compared multiple surveillance streams and found that no single indicator was sufficient to capture the full dynamics of COVID-19. Rather, a complementary, multichannel approach—combining hospital, wastewater, digital data with meteorological variables—provided the most robust situational awareness and generally showed contemporaneous alignment with short lead potential up to 7 days. Integrating these streams allowed us to exploit their distinct temporal properties and public health uses, supporting timely detection, interpretation, and response to evolving transmission patterns.

Wastewater surveillance emerged as a particularly valuable early signal. Our time-series models, which controlled for key meteorological confounders, demonstrated that N gene concentration and positivity rate were significant predictors of reported cases, with a lead time of 0‐7 days. These associations were relatively robust to short-term weather variation, consistent with wastewater’s capacity to capture community-level viral circulation, including asymptomatic and presymptomatic infections, before clinical case ascertainment [25-27]. While its rolling correlation exhibited variation (N gene concentration, median 0.585, IQR: 0.214‐0.766), this likely reflected its sensitivity to rapid shifts in transmission dynamics that case-based reporting only later captured. Our study also indicated that the wastewater monitoring of SARS-CoV-2 positivity rate and concentration exhibited moderate correlations with reported cases (all ρ>0.5).

Hospital-based NAAT positive rate from sentinel hospitals proved to be the most stable and reliable indicator for real-time tracking (median 0.534, IQR 0.394‐0.724; 58% of rolling windows ρ>0.5), showing peak association at lag 0 days. The substantial IQR of correlation coefficients suggested a lack of robustness in the associations between most variables and reported cases, indicating significant fluctuation in the correlation intensity over time. The high sensitivity of clinic-based NAAT testing highlighted its critical role in identifying infected individuals and accurately tracing the trajectory of the pandemic [28,29]. Our results indicated that the correlation between fever clinic visits and reported cases exhibited significant fluctuation over time (IQR –0.285 to 0.531). As the pandemic progresses and vaccination campaigns are rolled out, the symptoms of COVID-19 infections have diverged from the peak pandemic period [30,31]. In addition, in the postpandemic era, the prevalence of influenza may also impact the number of consultations for patients with COVID-19 [32].

Notably, our study added to the evidence on digital surveillance systems by specifically evaluating China’s Baidu Index data. Unlike some prior studies based on other platforms, which showed limited correlation with case counts (ρ=0.04‐0.08) [33], our analysis revealed that Baidu search metrics for COVID-19–related terms exhibited meaningful correlations with reported cases. Using a validated 7-day moving window [34], digital surveillance data provided a proxy for public awareness and health-seeking behavior, offering real-time insight into public perception and sentiment during outbreaks [35,36]. Several terms demonstrated intermediate temporal stability over a 60-day window (eg, BSI6 44.5% and BSI7 30.2%), supporting their use as indicators of behavioral intent. However, their temporal variability meant that they might be interpreted alongside clinical and wastewater data to mitigate false positives from media-driven or non–outbreak-related trends. Digital surveillance from Baidu showed heterogeneous performance and should primarily be treated as a behavioral and awareness signal rather than a direct proxy for case burden. Name-based queries (terms referring to COVID-19 or SARS-CoV-2) exhibited modest positive associations with reported cases, while symptom-based queries (terms for fever, cough, etc) were generally weaker and sometimes showed neutral or negative associations. Meteorological conditions and seasonal behaviors could modify these search patterns (eg, warmer weather or influenza seasonality could alter health-seeking and search behavior). Thus, digital indices were best seen as reflecting population concern and potential care-seeking. Consequently, we recommend using Baidu metrics as an early behavioral indicator. When corroborated by wastewater and NAAT data, they could help prioritize investigations, rather than being used to infer changes in actual community incidence.

Meteorological signals provide contextual information but do not demonstrate robust independent predictive power in multivariable models. Most studies have demonstrated negative correlations between temperature, humidity, and COVID-19 cases, but some have contested the extent of the influence of meteorological factors on the epidemic [37-41]. In this study, bivariate analyses showed mixed associations between meteorological variables and reported cases (positive associations with temperature, absolute humidity, visibility; negative association with air pressure), but these associations were substantially attenuated in multivariable time-series models that included surveillance streams. This aligned with the consensus that meteorological variables alone are insufficient to curb COVID-19 resurgence without concomitant public health interventions [42,43]. Meteorological indicators exhibited low rolling-window stability, with many showing a low proportion of windows where ρ exceeded 0.5. This implied that their primary use was more contextual, helping to interpret seasonal variations or behaviorally mediated changes, rather than functioning as reliable standalone predictors.

Data from diverse surveillance channels allows for scientific and rapid characterization of epidemiology, trend tracking, and outbreak prediction [44]. Drawing lessons from the COVID-19 pandemic, it is imperative to establish routine surveillance for infectious diseases. No single surveillance system can perfectly fulfill all information needs. Our findings supported a sequential, multilayered surveillance strategy: wastewater monitoring can serve as an early alert, digital indices can signal shifts in public concern and health-seeking that may precede care-seeking, and NAAT-based hospital surveillance provides confirmation and real-time tracking. Meteorological monitoring adds contextual information that can refine interpretation, particularly of behavioral signals, but is insufficient as a standalone predictor. During the year after policy relaxation, the city experienced 3 peaks in reported cases, with the largest wave from mid-February to late May 2024. By the end of the study period, SARS-CoV-2 showed patterns consistent with endemic circulation in the region, and integrated surveillance streams captured the rise and fall of transmission and informed timely public health action.

### Limitations

Our study had several limitations. First, taking COVID-19–reported cases as the benchmark might lead to an underestimation of the actual case count. In this study, we used the local CDC’s Information System for Disease Control and Prevention (CISDCP) to obtain the daily reported cases of COVID-19, which enabled us to gain the endemic situation to the greatest extent. However, since some COVID-19–infected individuals were asymptomatic and would not seek medical treatment without mandatory testing, our research might underestimate the actual number of infected people. Second, reported cases served as an operational benchmark but likely underestimated the actual incidence. Verification bias from variable care-seeking behavior and differential testing access (by time, locality, and population subgroup) might alter the relationship between true infections and reported cases. Such bias could affect correlations and lag estimates. To better address this, we recommend using a multichannel surveillance approach that combines wastewater and digital indicators, as well as representative serosurveys or adjustments for testing effort, to identify the trends. Nevertheless, it is imperative for future studies to incorporate hospitalization statistics to effectively and promptly monitor the severity of emerging infectious diseases. In addition, we conducted a short-term evaluation of the surveillance systems in only 1 city, and we did not consider the population heterogeneity and geographical heterogeneity. Future research should undertake more in-depth regional studies, adjust for covariates, and use multichannel surveillance systems to develop more accurate prediction models for infectious disease responses.

### Conclusions

In the postpandemic era, effective endemic preparedness requires a hybrid surveillance architecture that combines traditional clinical testing with environmental and digital streams. Wastewater and NAAT surveillance together provide timely, complementary signals of community transmission, while syndromic and internet-derived indicators offer rapid insight into health care burden and public behavior. Meteorological data contribute important context, but are insufficient alone. The optimal mix of systems might vary depending on local resources, technological infrastructure, and cultural factors, but should invariably include multiple complementary data streams.

Future research should focus on integrating multichannel data, expanding geographic scope, and incorporating hospitalization and pathogen-specific signals to strengthen early warning systems and public health decision-making. The integrated strategy will be key to enhancing the effectiveness of public health responses to respiratory pathogens.

## Supplementary material

10.2196/70232 Multimedia Appendix 1 Description of data from different surveillance systems.

10.2196/70232 Multimedia Appendix 2 Detailed explanation of the process used to gather Baidu Search Index data.

10.2196/70232 Multimedia Appendix 3 The reported cases of COVID-19 and data from different surveillance systems every day between April 1, 2023, and June 30, 2024.

10.2196/70232 Multimedia Appendix 4 Correlation analysis between different surveillance systems.

10.2196/70232 Multimedia Appendix 5 Temporal Lead Effects of different surveillance systems.

## Abbreviations

BSI: Baidu Search Index
CDC: Centers for Disease Control and Prevention
CISDCP: Information System for Disease Control and Prevention
DLNM: distributed lag nonlinear model
NAAT: nucleic acid amplification test
NDRIS: National Notifiable Disease Reporting Information System
