# Supplementary material for: Comparative Performance of Wastewater, Clinical, and Digital Surveillance Indicators for COVID-19 Monitoring in Routine Practice: Retrospective Observational Study
Source: J Med Internet Res. 2025 Nov 6;27:e70232. doi: 10.2196/70232 (PMC12592968; doi:10.2196/70232)
Supplement: Multimedia Appendix 1 [file jmir-v27-e70232-s001.docx]

**Table 1**. **Different surveillance systems and dataset descriptions**

| Surveillance systems and datasets | Description | Timeliness | Date range |
| --- | --- | --- | --- |
| **Operational benchmark** |  |  |  |
| case report | Daily Reported Cases | 1 day | April 1, 2023- June 30, 2024 |
| **Comparator surveillance systems** |  |  |  |
| Hospital Surveillance System |  |  |  |
| visits | Fever Clinic Visits | 1 day | April 1, 2023- June 30, 2024 |
| positive_rate | Positive Rate of Nucleic Acid Amplification Test | 1 day | April 1, 2023- June 30, 2024 |
| Wastewater Surveillance System |  |  |  |
| N gene concentration | Concentration of Nucleocapsid genome copies | Twice per week | April 1, 2023- June 30, 2024 |
| N gene positive rate | Positive rate of Nucleocapsid gene | Twice per week | April 1, 2023- June 30, 2024 |
| ORF1ab gene concentration | Concentration of Open Reading Frame 1ab genome copies | Twice per week | April 1, 2023- June 30, 2024 |
| ORF1ab gene positive rate | Positive rate of Open Reading Frame 1ab gene | Twice per week | April 1, 2023- June 30, 2024 |
| Meteorological Surveillance System |  |  |  |
| Tmean | Mean temperature | Every 3h per day | Mar 1, 2023- June 30, 2024 |
| Pmean | Mean air pressure | Every 3h per day | Mar 1, 2023- June 30, 2024 |
| RHmean | Mean relative humidity | Every 3h per day | Mar 1, 2023- June 30, 2024 |
| AHmean | Mean absolute humidity | Every 3h per day | Mar 1, 2023- June 30, 2024 |
| WSmean | Mean wind speed | Every 3h per day | Mar 1, 2023- June 30, 2024 |
| VISmean | Mean visibility | Every 3h per day | Mar 1, 2023- June 30, 2024 |
| PRCPmean | Mean precipitation | Every 3h per day | Mar 1, 2023- June 30, 2024 |
| Internet Search Engine System |  |  |  |
| Symptom-related |  |  |  |
| BSI1 | BSI1, Baidu search rank for “fever” | 1 day | Mar 1, 2023- June 30, 2024 |
| BSI2 | BSI2, Baidu search rank for “cough” | 1 day | Mar 1, 2023- June 30, 2024 |
| BSI3 | BSI3, Baidu search rank for “sore throat” | 1 day | Mar 1, 2023- June 30, 2024 |
| BSI4 | BSI4, Baidu search rank for “weakness” | 1 day | Mar 1, 2023- June 30, 2024 |
| BSI5 | BSI5, Baidu search rank for “diarrhea” | 1 day | Mar 1, 2023- June 30, 2024 |
| Name-related |  |  |  |
| BSI6 | BSI6, Baidu search rank for “COVID-19” | 1 day | Mar 1, 2023- June 30, 2024 |
| BSI7 | BSI7, Baidu search rank for “novel coronavirus” | 1 day | Mar 1, 2023- June 30, 2024 |
| BSI8 | BSI8, Baidu search rank for “COVID-19 pneumonia” | 1 day | Mar 1, 2023- June 30, 2024 |
| BSI9 | BSI9, Baidu search rank for “novel coronavirus pneumonia” | 1 day | Mar 1, 2023- June 30, 2024 |
| BSI10 | BSI10, Baidu search rank for “Omicron” | 1 day | Mar 1, 2023- June 30, 2024 |

**Table 2**. **Data Availability and Completeness**

| Surveillance systems and datasets | Total Study Days | Days with Complete Data (%) | Days with Missing Data (%) |
| --- | --- | --- | --- |
| **Operational benchmark** |  |  |  |
| case report | 457 | **409 (89.50)** | 48 (10.50) |
| **Comparator surveillance systems** |  |  |  |
| Hospital Surveillance System |  |  |  |
| visits | 457 | 457 (100) | 0 (0) |
| positive_rate | 457 | 457 (100) | 0 (0) |
| Wastewater Surveillance System |  |  |  |
| N gene concentration | 457 | 457 (100) | 0 (0) |
| N gene positive rate | 457 | 457 (100) | 0 (0) |
| ORF1ab gene concentration | 457 | 457 (100) | 0 (0) |
| ORF1ab gene positive rate | 457 | 457 (100) | 0 (0) |
| Meteorological Surveillance System |  |  |  |
| Tmean | **488** | **488 (100)** | 0 (0) |
| Pmean | **488** | **488 (100)** | 0 (0) |
| RHmean | **488** | **488 (100)** | 0 (0) |
| AHmean | **488** | **488 (100)** | 0 (0) |
| WSmean | **488** | **488 (100)** | 0 (0) |
| VISmean | **488** | **488 (100)** | 0 (0) |
| PRCPmean | **488** | **488 (100)** | 0 (0) |
| Internet Search Engine System |  |  |  |
| Symptom-related |  |  |  |
| BSI1 | **488** | **488 (100)** | 0 (0) |
| BSI2 | **488** | **488 (100)** | 0 (0) |
| BSI3 | **488** | **488 (100)** | 0 (0) |
| BSI4 | **488** | **488 (100)** | 0 (0) |
| BSI5 | **488** | **488 (100)** | 0 (0) |
| Name-related |  |  |  |
| BSI6 | **488** | **488 (100)** | 0 (0) |
| BSI7 | **488** | **488 (100)** | 0 (0) |
| BSI8 | **488** | **488 (100)** | 0 (0) |
| BSI9 | **488** | **488 (100)** | 0 (0) |
| BSI10 | **488** | **488 (100)** | 0 (0) |
