# Supplementary material for: Comparative Performance of Wastewater, Clinical, and Digital Surveillance Indicators for COVID-19 Monitoring in Routine Practice: Retrospective Observational Study
Source: J Med Internet Res. 2025 Nov 6;27:e70232. doi: 10.2196/70232 (PMC12592968; doi:10.2196/70232)
Supplement: Multimedia Appendix 2 [file jmir-v27-e70232-s002.docx]

1. Setup: Install the ‘qdata’ package and necessary libraries (‘pandas’, ‘openpyxl’). Prepare a list of keywords related to the research topic and obtain a valid Baidu cookie (BDUSS) for API authentication.
2. Authentication: Use the BDUSS cookie to authenticate access to the Baidu Index API, which is essential for retrieving search data.
3. Parameter Configuration: Define the start and end dates for the data collection period, specify the region of interest using the corresponding area code (refer to the provided PROVINCE_CODE and CITY_CODE dictionaries), and set the type of search data to collect (e.g., PC, mobile, or aggregated).
4. Data Collection Workflow: Utilize the ‘get_search_index’ function from the ‘qdata.baidu_index’ module, passing in the keywords, date range, cookies, and area code. The ‘split_keywords’ function is employed to process keywords in batches, adhering to API limitations. The retrieved data is collected in a dictionary format, with each entry containing the keyword, search type, index value, and date.
5. Data Processing: Convert the collected data into a pandas ‘DataFrame’ for easier manipulation and analysis. The ‘DataFrame’ is then exported to an Excel file, with columns representing the keyword, search type, index value, and date.
6. Location and Keyword Details: The PROVINCE_CODE and CITY_CODE dictionaries map specific region names to their respective codes, ensuring accurate data collection for the desired geographic areas. The keywords used in the study are selected based on their relevance to the research objectives and are listed in the code for transparency.
7. Partial code:

import qdata

import openpyxl

import time

from qdata.baidu_index import get_search_index

from qdata.baidu_index.common import split_keywords

import pandas as pd

import os

# Input the keywords you want to search, with a maximum of five at a time

keywords_list = [['word1'], ['word2'], ['word3'], ['word4'], ['word5']]

# Set COOKIE, format 'BDUSS=cookie value'

cookies = "BDUSS=……" #'BDUSS=cookie value'

# Set up an empty dictionary data for storing data, which will be used to create an Excel table with column names keyword, type, index, date

data = {'keyword': [], 'type': [], 'index': [], 'date': []}

# Input the city or province code, 911 represents Beijing, refer to the following for more city codes

area_code = 911

# Split keywords and loop to write the crawled data into data

for keywords in split_keywords(keywords_list):

    # get_search_index is the key function, you can check it for more data items

    for index in get_search_index(

            keywords_list=keywords,

            start_date='2023-01-01',

            end_date='2024-07-30',

            cookies=cookies,

            area=area_code

            ):

        data['keyword'].append(index['keyword'])

        data['type'].append(index['type'])

        data['index'].append(index['index'])

        data['date'].append(index['date'])

    time.sleep(5)

# Convert data to DataFrame for easy export to Excel

# Different parameters for type: type_all represents PC + mobile; type_pc represents PC data; type_wise represents mobile datadata_final = pd.DataFrame(data)

data_final.to_excel("baidu_index_result0911.xlsx")

current_directory = os.getcwd()

print("Current working directory", current_directory)

# Province codes (partial)

PROVINCE_CODE = {'Shandong': '901', 'Guizhou': '902', 'Jiangxi': '903', 'Chongqing': '904', 'Inner Mongolia': '905', 'Hubei': '906', 'Liaoning': '907', 'Hunan': '908',

'Fujian': '909', 'Shanghai': '910', 'Beijing': '911'}

# City codes (partial)

CITY_CODE = {'Suqian': '172', 'Wuhan': '28', 'Huangshi': '30', 'Jingzhou': '31', 'Xiangyang': '32', 'Huanggang': '33', 'Jingmen': '34', 'Yichang': '35', 'Shiyan': '36', 'Suizhou': '37', 'Enshi': '38', 'Ezhou': '39', 'Xianning': '40', 'Xiaogan': '41', 'Xiantao': '42', 'Tianmen': '73', 'Qianjiang': '74','Shennongjia': '687', 'Hangzhou': '138', 'Lishui': '134', 'Jinhua': '135', 'Wenzhou': '149', 'Taizhou': '287', 'Quzhou': '288', 'Ningbo': '289'}
