# Supplementary material for: Comparative Performance of Wastewater, Clinical, and Digital Surveillance Indicators for COVID-19 Monitoring in Routine Practice: Retrospective Observational Study
Source: J Med Internet Res. 2025 Nov 6;27:e70232. doi: 10.2196/70232 (PMC12592968; doi:10.2196/70232)
Supplement: Multimedia Appendix 3 [file jmir-v27-e70232-s003.docx]

**Figure S1.**

**
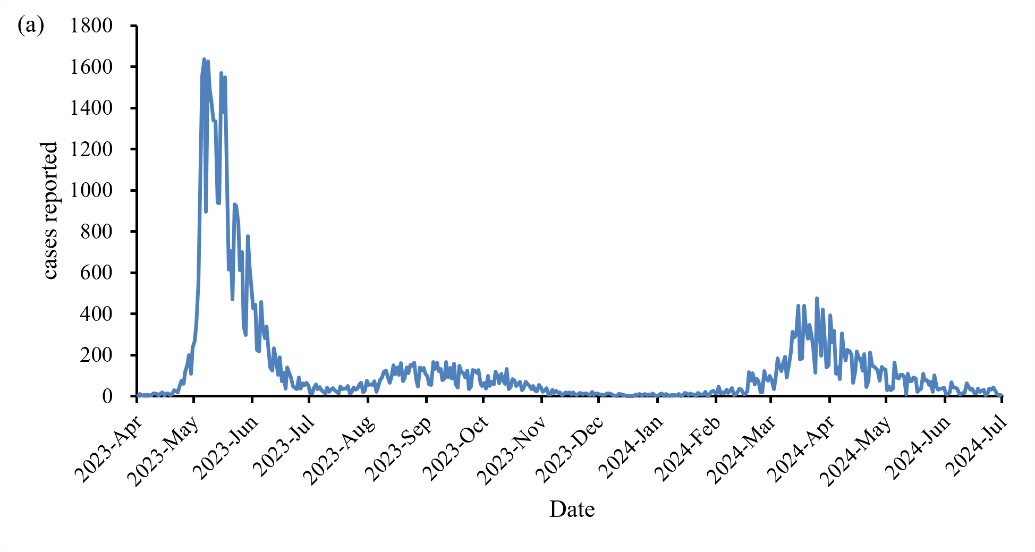
**

**
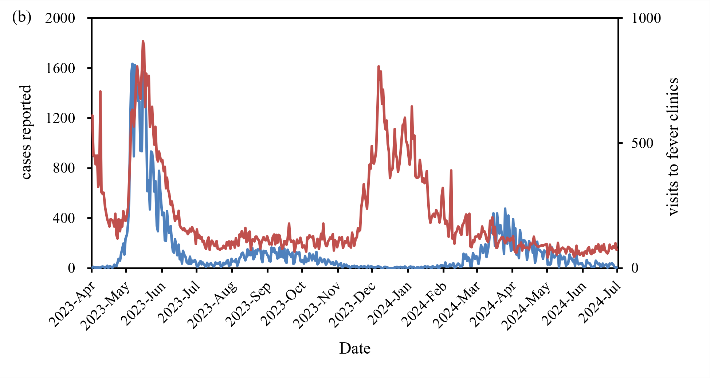

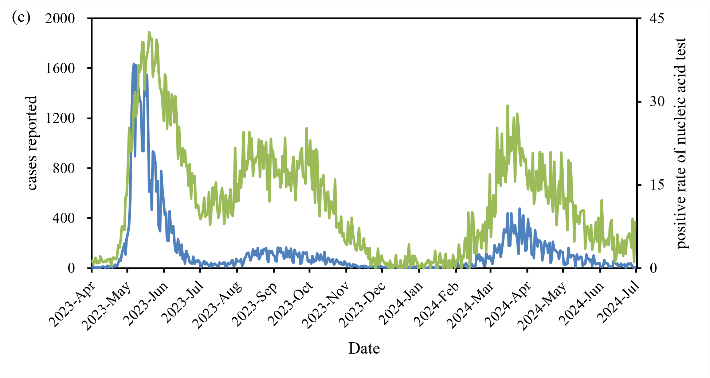
**

**
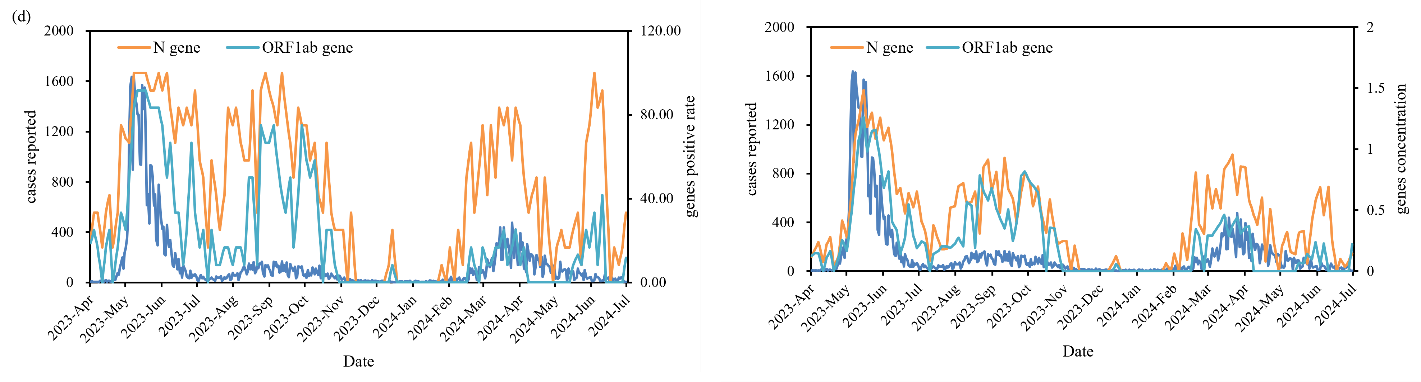
**

**
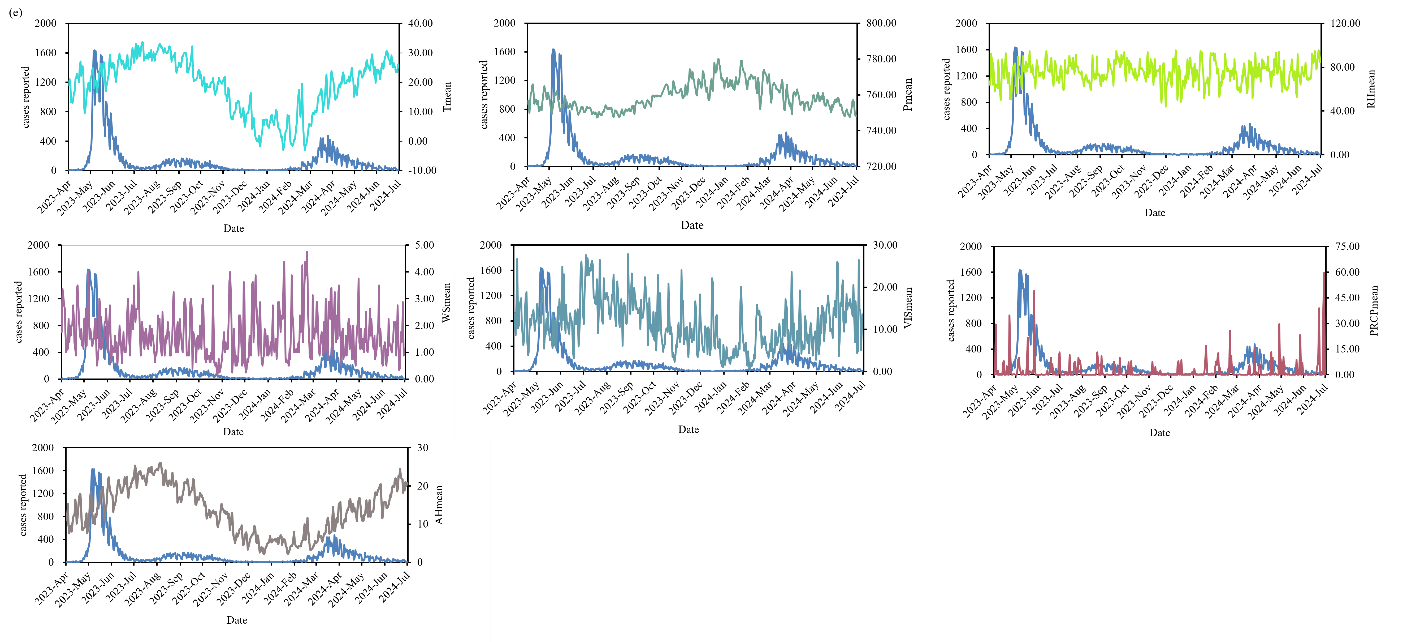
**

**
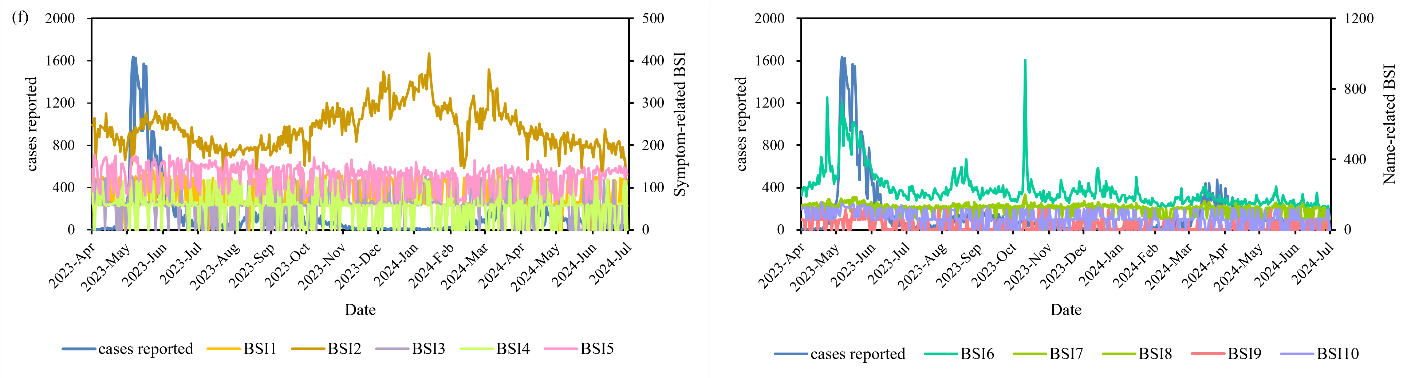
**

(a) case reported; (b) visits; (c) positive_rate; (d) wastewater surveillance system; (e) meteorological surveillance system; (f) Internet search engine system.
