# Supplementary material for: Comparative Performance of Wastewater, Clinical, and Digital Surveillance Indicators for COVID-19 Monitoring in Routine Practice: Retrospective Observational Study
Source: J Med Internet Res. 2025 Nov 6;27:e70232. doi: 10.2196/70232 (PMC12592968; doi:10.2196/70232)
Supplement: Multimedia Appendix 4 [file jmir-v27-e70232-s004.docx]

**Table 1. Correlation analysis between different surveillance systems for moving 60-day window between April, 2023 to June, 2024**

| Surveillance systems and datasets | Median | IQR |
| --- | --- | --- |
| **Notifiable Disease Reporting Information System** |  |  |
| cases reported | 1 | 1 - 1 |
| **Comparator surveillance systems** |  |  |
| **Hospital Surveillance System** |  |  |
| visits | 0.252 | -0.285 - 0.531 |
| positive_rate | 0.534 | 0.394 - 0.724 |
| **Wastewater Surveillance System** |  |  |
| N gene concentration | 0.530 | 0.222 - 0.742 |
| N gene positive rate | 0.585 | 0.214 - 0.766 |
| ORF1ab gene concentration | 0.467 | 0.080 - 0.729 |
| ORF1ab gene positive rate | 0.522 | -0.005 - 0.745 |
| **Meteorological Surveillance System** |  |  |
| Tmean | 0.016 | -0.246 - 0.412 |
| Pmean | -0.019 | -0.268 - 0.221 |
| RHmean | -0.032 | -0.185 - 0.126 |
| AHmean | -0.001 | -0.363 - 0.427 |
| WSmean | 0.001 | -0.106 - 0.134 |
| VISmean | 0.027 | -0.175 - 0.205 |
| PRCPmean | -0.019 | -0.115 - 0.101 |
| **Internet Search Engine System** |  |  |
| BSI1, Baidu search rank for “fever” | 0.077 | -0.024 - 0.282 |
| BSI2, Baidu search rank for “cough” | 0.149 | -0.315 - 0.423 |
| BSI3, Baidu search rank for “sore throat” | 0.053 | -0.034 - 0.206 |
| BSI4, Baidu search rank for “weakness” | 0.093 | -0.027 - 0.190 |
| BSI5, Baidu search rank for “diarrhea” | 0.188 | 0.075 - 0.293 |
| BSI6, Baidu search rank for “COVID-19” | 0.475 | 0.192 - 0.685 |
| BSI7, Baidu search rank for “novel coronavirus” | 0.355 | 0.063 - 0.538 |
| BSI8, Baidu search rank for “COVID-19 pneumonia” | 0.229 | 0.095 - 0.387 |
| BSI9, Baidu search rank for “novel coronavirus pneumonia” | 0.013 | -0.087 - 0.128 |
| BSI10, Baidu search rank for “Omicron” | 0.147 | 0.013 - 0.285 |

**Figure 1: Spearman correlation analyses between different surveillance systems and reported cases with a moving 60-day window between April 1, 2023 to June 30, 2024**


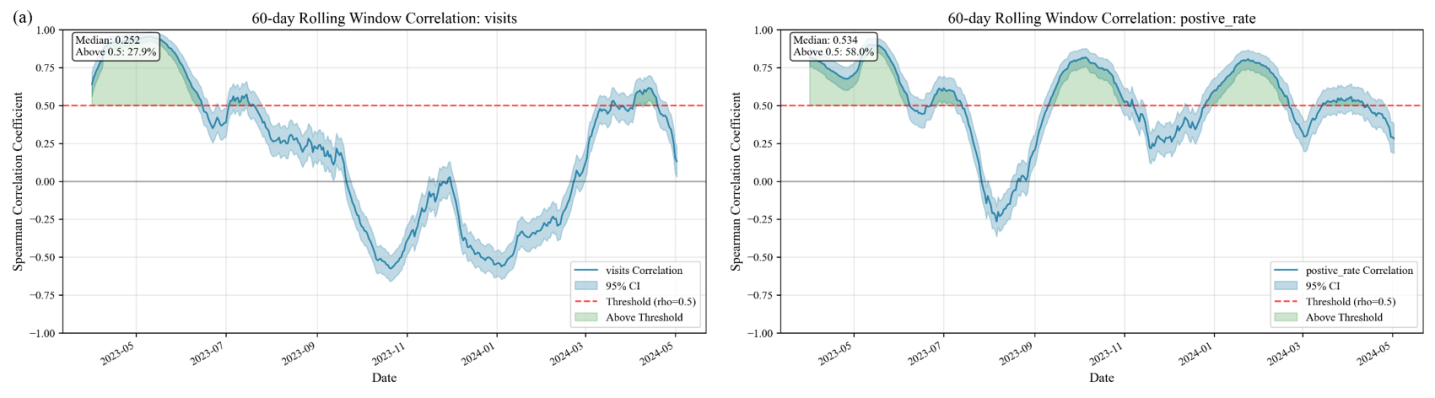


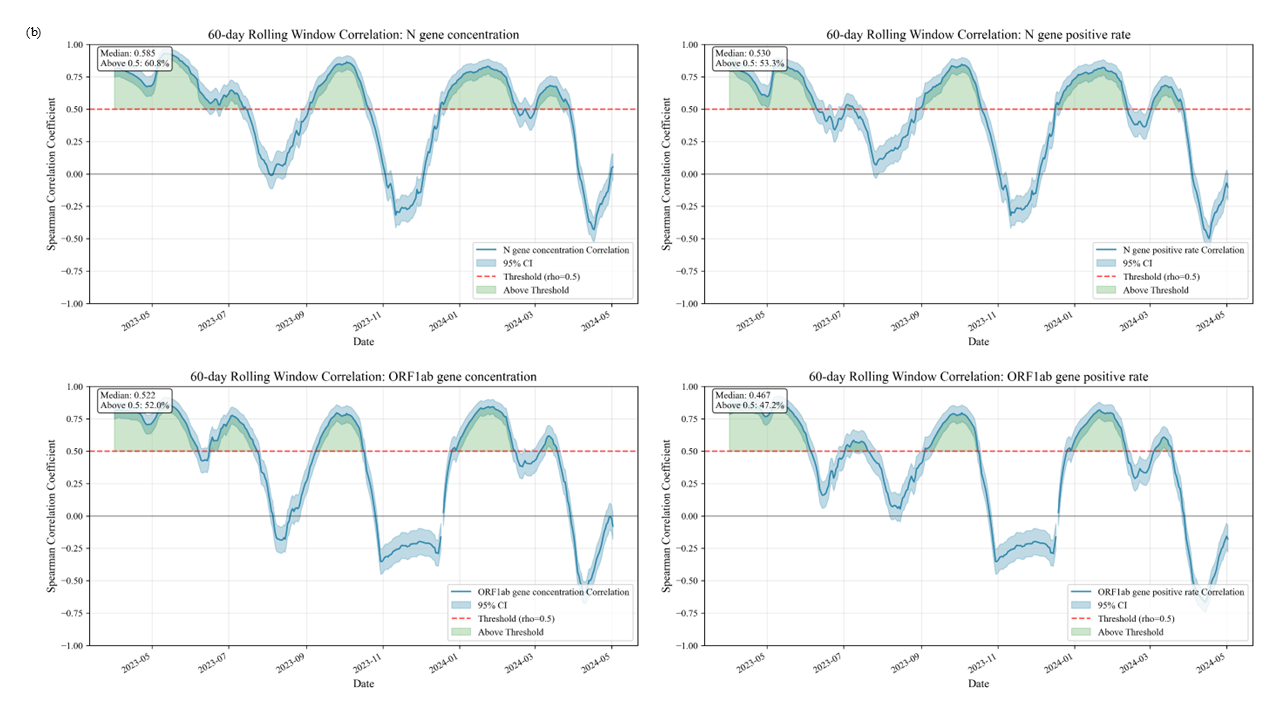


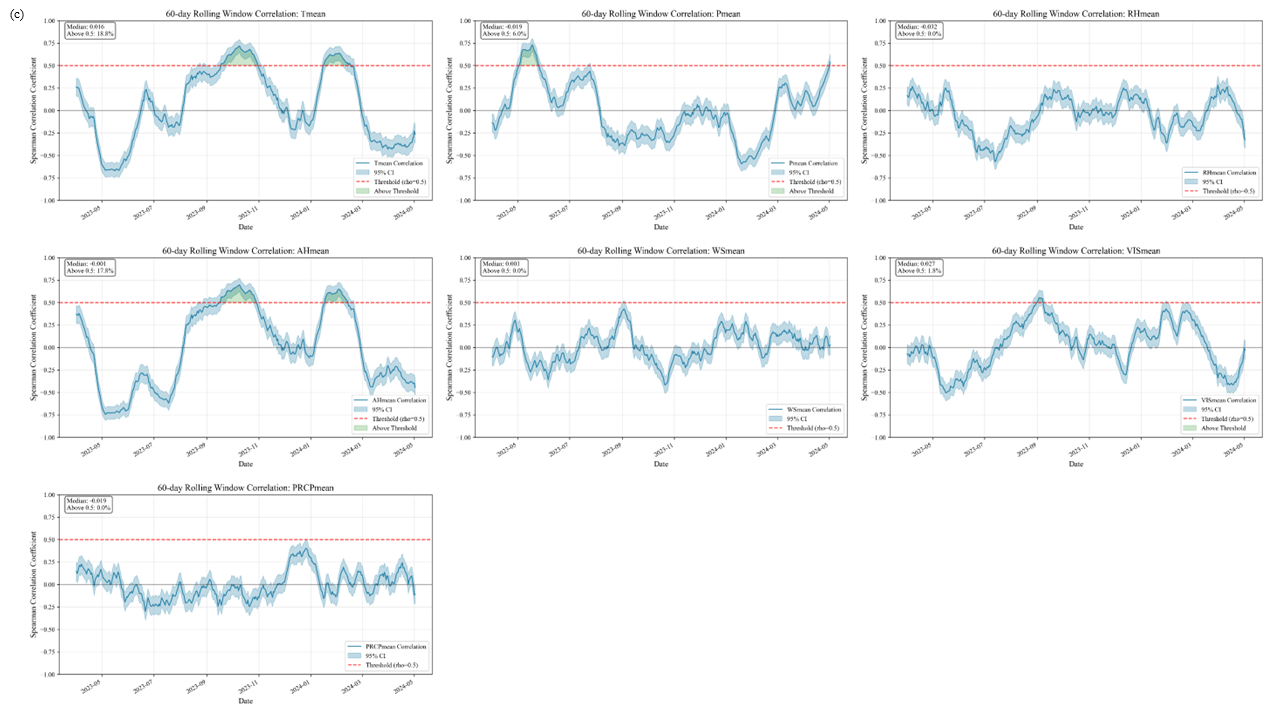


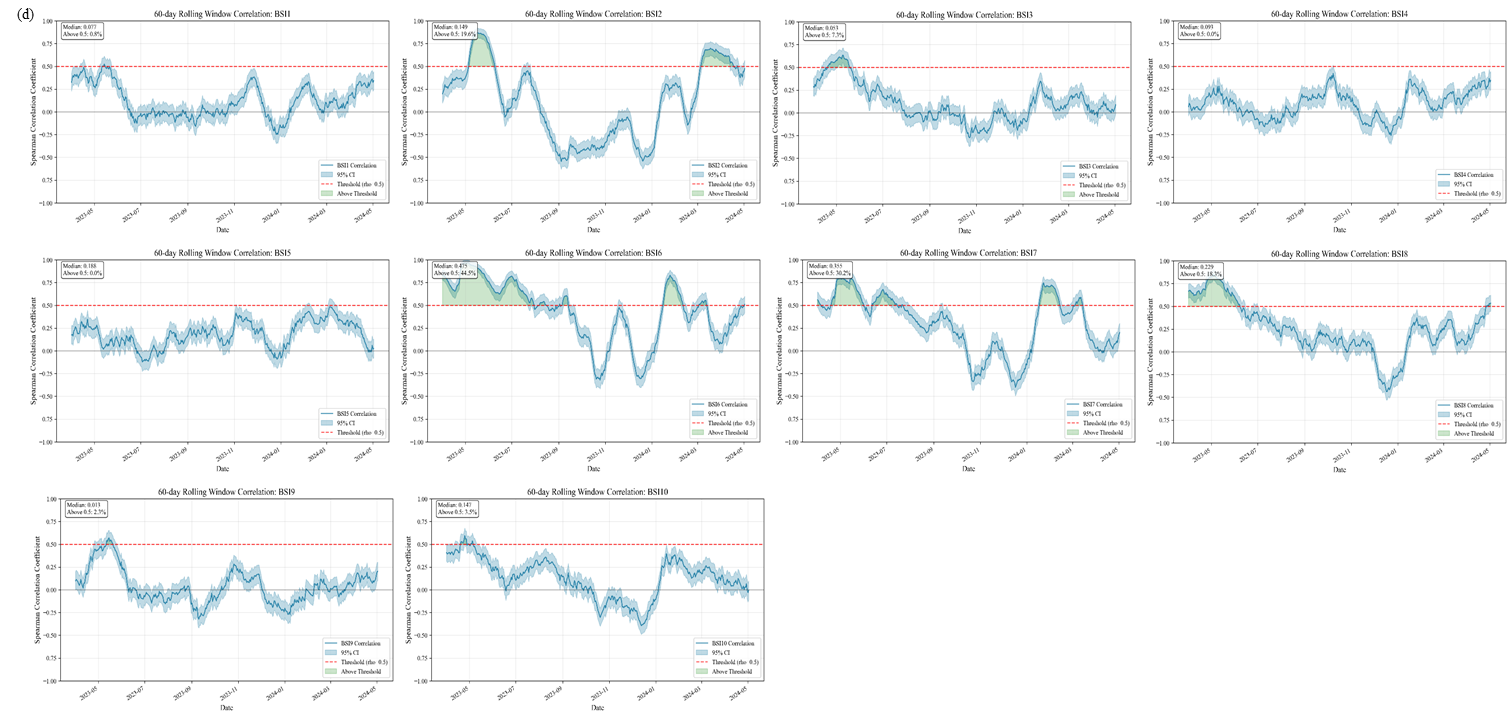


(a) hospital surveillance system; (b) wastewater surveillance system; (c) meteorological surveillance system; (d) Internet search engine system.

**Table 2. Spearman correlation analysis between different surveillance systems and reported cases with a 7-day lag before and after between April, 2023 to June, 2024**

| Surveillance systems and datasets | Lag days | Coefficient (95%CI) | *P* value |
| --- | --- | --- | --- |
| **Hospital Surveillance System** |  |  |  |
| visits | -7 | -0.143 (-0.231 to -0.052) | **.002** |
|  | -6 | -0.152 (-0.240 to -0.062) | **.001** |
|  | -5 | -0.142 (-0.230 to -0.052) | **.002** |
|  | -4 | -0.134 (-0.222 to -0.044) | **.004** |
|  | -3 | -0.133 (-0.221 to -0.043) | **.004** |
|  | -2 | -0.127 (-0.216 to -0.037) | **.006** |
|  | -1 | -0.098 (-0.187 to -0.008) | **.04** |
|  | 1 | -0.114 (-0.203 to -0.024) | **.01** |
|  | 2 | -0.110 (-0.199 to -0.020) | **.02** |
|  | 3 | -0.121 (-0.209 to -0.030) | **.01** |
|  | 4 | -0.127 (-0.215 to -0.037) | **.007** |
|  | 5 | -0.123 (-0.211 to -0.032) | **.009** |
|  | 6 | -0.105 (-0.194 to -0.014) | **.03** |
|  | 7 | -0.104 (-0.193 to -0.013) | **.03** |
| positive_rate | -7 | 0.827 (0.796 to 0.854) | **<.001** |
|  | -6 | 0.843 (0.815 to 0.868) | **<.001** |
|  | -5 | 0.845 (0.817 to 0.869) | **<.001** |
|  | -4 | 0.839 (0.810 to 0.864) | **<.001** |
|  | -3 | 0.853 (0.826 to 0.876) | **<.001** |
|  | -2 | 0.857 (0.831 to 0.880) | **<.001** |
|  | -1 | 0.849 (0.822 to 0.872) | **<.001** |
|  | 1 | 0.839 (0.810 to 0.864) | **<.001** |
|  | 2 | 0.825 (0.794 to 0.852) | **<.001** |
|  | 3 | 0.827 (0.796 to 0.854) | **<.001** |
|  | 4 | 0.806 (0.772 to 0.836) | **<.001** |
|  | 5 | 0.808 (0.774 to 0.837) | **<.001** |
|  | 6 | 0.786 (0.749 to 0.819) | **<.001** |
|  | 7 | 0.769 (0.729 to 0.804) | **<.001** |
| **Wastewater Surveillance System** |  |  |  |
| N gene positive rate | -7 | 0.676 (0.624 to 0.723) | **<.001** |
|  | -6 | 0.682 (0.630 to 0.727) | **<.001** |
|  | -5 | 0.682 (0.630 to 0.728) | **<.001** |
|  | -4 | 0.684 (0.633 to 0.730) | **<.001** |
|  | -3 | 0.691 (0.640 to 0.735) | **<.001** |
|  | -2 | 0.697 (0.648 to 0.741) | **<.001** |
|  | -1 | 0.700 (0.650 to 0.743) | **<.001** |
|  | 1 | 0.697 (0.648 to 0.741) | **<.001** |
|  | 2 | 0.694 (0.644 to 0.738) | **<.001** |
|  | 3 | 0.693 (0.643 to 0.737) | **<.001** |
|  | 4 | 0.693 (0.642 to 0.737) | **<.001** |
|  | 5 | 0.690 (0.639 to 0.735) | **<.001** |
|  | 6 | 0.679 (0.626 to 0.725) | **<.001** |
|  | 7 | 0.662 (0.607 to 0.710) | **<.001** |
| N gene concentration | -7 | 0.752 (0.709 to 0.789) | **<.001** |
|  | -6 | 0.761 (0.720 to 0.797) | **<.001** |
|  | -5 | 0.764 (0.724 to 0.800) | **<.001** |
|  | -4 | 0.764 (0.723 to 0.799) | **<.001** |
|  | -3 | 0.766 (0.726 to 0.801) | **<.001** |
|  | -2 | 0.775 (0.736 to 0.808) | **<.001** |
|  | -1 | 0.777 (0.738 to 0.810) | **<.001** |
|  | 1 | 0.774 (0.735 to 0.808) | **<.001** |
|  | 2 | 0.773 (0.734 to 0.807) | **<.001** |
|  | 3 | 0.768 (0.728 to 0.802) | **<.001** |
|  | 4 | 0.763 (0.722 to 0.798) | **<.001** |
|  | 5 | 0.758 (0.717 to 0.794) | **<.001** |
|  | 6 | 0.745 (0.701 to 0.782) | **<.001** |
|  | 7 | 0.729 (0.683 to 0.769) | **<.001** |
| ORF1ab gene positive rate | -7 | 0.571 (0.507 to 0.630) | **<.001** |
|  | -6 | 0.573 (0.509 to 0.631) | **<.001** |
|  | -5 | 0.583 (0.519 to 0.640) | **<.001** |
|  | -4 | 0.583 (0.520 to 0.640) | **<.001** |
|  | -3 | 0.580 (0.517 to 0.638) | **<.001** |
|  | -2 | 0.585 (0.522 to 0.642) | **<.001** |
|  | -1 | 0.582 (0.519 to 0.639) | **<.001** |
|  | 1 | 0.578 (0.514 to 0.635) | **<.001** |
|  | 2 | 0.579 (0.515 to 0.636) | **<.001** |
|  | 3 | 0.575 (0.510 to 0.632) | **<.001** |
|  | 4 | 0.569 (0.504 to 0.627) | **<.001** |
|  | 5 | 0.561 (0.495 to 0.620) | **<.001** |
|  | 6 | 0.547 (0.480 to 0.608) | **<.001** |
|  | 7 | 0.533 (0.464 to 0.595) | **<.001** |
| ORF1ab gene concentration | -7 | 0.594 (0.532 to 0.650) | **<.001** |
|  | -6 | 0.601 (0.540 to 0.656) | **<.001** |
|  | -5 | 0.617 (0.557 to 0.670) | **<.001** |
|  | -4 | 0.630 (0.571 to 0.681) | **<.001** |
|  | -3 | 0.643 (0.587 to 0.693) | **<.001** |
|  | -2 | 0.663 (0.609 to 0.711) | **<.001** |
|  | -1 | 0.667 (0.614 to 0.715) | **<.001** |
|  | 1 | 0.661 (0.607 to 0.709) | **<.001** |
|  | 2 | 0.657 (0.602 to 0.705) | **<.001** |
|  | 3 | 0.654 (0.599 to 0.703) | **<.001** |
|  | 4 | 0.657 (0.602 to 0.706) | **<.001** |
|  | 5 | 0.660 (0.606 to 0.708) | **<.001** |
|  | 6 | 0.652 (0.597 to 0.702) | **<.001** |
|  | 7 | 0.638 (0.581 to 0.689) | **<.001** |
| **Meteorological Surveillance System** |  |  |  |
| Tmean | -7 | 0.352 (0.269 to 0.429) | **<.001** |
|  | -6 | 0.347 (0.264 to 0.425) | **<.001** |
|  | -5 | 0.348 (0.265 to 0.425) | **<.001** |
|  | -4 | 0.352 (0.270 to 0.429) | **<.001** |
|  | -3 | 0.358 (0.277 to 0.435) | **<.001** |
|  | -2 | 0.349 (0.267 to 0.426) | **<.001** |
|  | -1 | 0.348 (0.266 to 0.425) | **<.001** |
|  | 1 | 0.336 (0.253 to 0.414) | **<.001** |
|  | 2 | 0.328 (0.245 to 0.406) | **<.001** |
|  | 3 | 0.324 (0.240 to 0.403) | **<.001** |
|  | 4 | 0.314 (0.230 to 0.394) | **<.001** |
|  | 5 | 0.306 (0.222 to 0.386) | **<.001** |
|  | 6 | 0.304 (0.219 to 0.384) | **<.001** |
|  | 7 | 0.304 (0.219 to 0.384) | **<.001** |
| Pmean | -7 | -0.337 (-0.415 to -0.254) | **<.001** |
|  | -6 | -0.344 (-0.422 to -0.262) | **<.001** |
|  | -5 | -0.355 (-0.432 to -0.273) | **<.001** |
|  | -4 | -0.358 (-0.435 to -0.276) | **<.001** |
|  | -3 | -0.341 (-0.418 to -0.258) | **<.001** |
|  | -2 | -0.324 (-0.403 to -0.241) | **<.001** |
|  | -1 | -0.318 (-0.397 to -0.234) | **<.001** |
|  | 1 | -0.324 (-0.403 to -0.241) | **<.001** |
|  | 2 | -0.323 (-0.401 to -0.239) | **<.001** |
|  | 3 | -0.326 (-0.404 to -0.242) | **<.001** |
|  | 4 | -0.318 (-0.397 to -0.234) | **<.001** |
|  | 5 | -0.305 (-0.385 to -0.220) | **<.001** |
|  | 6 | -0.295 (-0.376 to -0.210) | **<.001** |
|  | 7 | -0.295 (-0.376 to -0.210) | **<.001** |
| RHmean | -7 | 0.071 (-0.020 to 0.161) | .13 |
|  | -6 | 0.091 (0.000 to 0.180) | .05 |
|  | -5 | 0.077 (-0.014 to 0.167) | .10 |
|  | -4 | 0.064 (-0.027 to 0.154) | .17 |
|  | -3 | 0.025 (-0.066 to 0.116) | .59 |
|  | -2 | 0.042 (-0.048 to 0.132) | .37 |
|  | -1 | 0.020 (-0.071 to 0.110) | .67 |
|  | 1 | 0.024 (-0.067 to 0.114) | .61 |
|  | 2 | 0.013 (-0.077 to 0.104) | .78 |
|  | 3 | 0.021 (-0.069 to 0.112) | .65 |
|  | 4 | 0.012 (-0.079 to 0.102) | .80 |
|  | 5 | 0.006 (-0.085 to 0.097) | .90 |
|  | 6 | -0.004 (-0.095 to 0.087) | .93 |
|  | 7 | 0.000 (-0.091 to 0.091) | .99 |
| AHmean | -7 | 0.353 (0.271 to 0.430) | **<.001** |
|  | -6 | 0.355 (0.273 to 0.432) | **<.001** |
|  | -5 | 0.352 (0.270 to 0.429) | **<.001** |
|  | -4 | 0.353 (0.271 to 0.430) | **<.001** |
|  | -3 | 0.347 (0.265 to 0.425) | **<.001** |
|  | -2 | 0.342 (0.260 to 0.420) | **<.001** |
|  | -1 | 0.335 (0.252 to 0.413) | **<.001** |
|  | 1 | 0.328 (0.244 to 0.406) | **<.001** |
|  | 2 | 0.319 (0.236 to 0.398) | **<.001** |
|  | 3 | 0.319 (0.235 to 0.398) | **<.001** |
|  | 4 | 0.308 (0.224 to 0.388) | **<.001** |
|  | 5 | 0.298 (0.213 to 0.379) | **<.001** |
|  | 6 | 0.289 (0.204 to 0.370) | **<.001** |
|  | 7 | 0.289 (0.204 to 0.371) | **<.001** |
| WSmean | -7 | -0.004 (-0.095 to 0.088) | .94 |
|  | -6 | 0.005 (-0.086 to 0.096) | .92 |
|  | -5 | 0.004 (-0.087 to 0.095) | .93 |
|  | -4 | 0.022 (-0.069 to 0.113) | .64 |
|  | -3 | 0.049 (-0.042 to 0.139) | .30 |
|  | -2 | 0.033 (-0.058 to 0.123) | .48 |
|  | -1 | 0.052 (-0.039 to 0.141) | .27 |
|  | 1 | 0.056 (-0.035 to 0.146) | .23 |
|  | 2 | 0.049 (-0.042 to 0.139) | .30 |
|  | 3 | 0.061 (-0.030 to 0.151) | .20 |
|  | 4 | 0.086 (-0.004 to 0.176) | .07 |
|  | 5 | 0.107 (0.016 to 0.196) | **.02** |
|  | 6 | 0.134 (0.043 to 0.222) | **.004** |
|  | 7 | 0.138 (0.047 to 0.226) | **.003** |
| VISmean | -7 | 0.163 (0.073 to 0.251) | **<.001** |
|  | -6 | 0.141 (0.051 to 0.229) | **.002** |
|  | -5 | 0.136 (0.046 to 0.224) | **.004** |
|  | -4 | 0.138 (0.048 to 0.226) | **.003** |
|  | -3 | 0.142 (0.052 to 0.230) | **.002** |
|  | -2 | 0.135 (0.045 to 0.223) | **.004** |
|  | -1 | 0.160 (0.070 to 0.247) | **.001** |
|  | 1 | 0.154 (0.065 to 0.241) | **.001** |
|  | 2 | 0.167 (0.078 to 0.254) | **<.001** |
|  | 3 | 0.153 (0.064 to 0.241) | **.001** |
|  | 4 | 0.152 (0.062 to 0.239) | **.001** |
|  | 5 | 0.160 (0.070 to 0.247) | **.001** |
|  | 6 | 0.195 (0.106 to 0.281) | **<.001** |
|  | 7 | 0.180 (0.090 to 0.266) | **<.001** |
| PRCPmean | -7 | 0.053 (-0.039 to 0.143) | .26 |
|  | -6 | 0.080 (-0.012 to 0.169) | .09 |
|  | -5 | 0.075 (-0.016 to 0.165) | .11 |
|  | -4 | 0.078 (-0.013 to 0.167) | .10 |
|  | -3 | 0.071 (-0.020 to 0.161) | .13 |
|  | -2 | 0.080 (-0.010 to 0.169) | .09 |
|  | -1 | 0.058 (-0.032 to 0.148) | .21 |
|  | 1 | 0.034 (-0.057 to 0.124) | .48 |
|  | 2 | 0.041 (-0.050 to 0.131) | .39 |
|  | 3 | 0.033 (-0.058 to 0.123) | .49 |
|  | 4 | 0.053 (-0.038 to 0.143) | .26 |
|  | 5 | 0.082 (-0.008 to 0.172) | .08 |
|  | 6 | 0.058 (-0.033 to 0.148) | .22 |
|  | 7 | 0.050 (-0.041 to 0.140) | .29 |
| **Internet Search Engine System** |  |  |  |
| BSI1 | -7 | 0.112 (0.021 to 0.201) | **.02** |
|  | -6 | 0.108 (0.018 to 0.197) | **.02** |
|  | -5 | 0.048 (-0.043 to 0.138) | .30 |
|  | -4 | 0.049 (-0.042 to 0.139) | .30 |
|  | -3 | 0.045 (-0.046 to 0.135) | .33 |
|  | -2 | 0.041 (-0.050 to 0.131) | .38 |
|  | -1 | 0.059 (-0.032 to 0.148) | .21 |
|  | 1 | 0.044 (-0.047 to 0.134) | .35 |
|  | 2 | 0.029 (-0.061 to 0.120) | .53 |
|  | 3 | -0.014 (-0.104 to 0.077) | .77 |
|  | 4 | -0.023 (-0.114 to 0.067) | .62 |
|  | 5 | -0.039 (-0.130 to 0.052) | .40 |
|  | 6 | -0.004 (-0.095 to 0.087) | .93 |
|  | 7 | 0.011 (-0.080 to 0.102) | .81 |
| BSI2 | -7 | -0.200 (-0.286 to -0.111) | **<.001** |
|  | -6 | -0.182 (-0.269 to -0.093) | **<.001** |
|  | -5 | -0.197 (-0.282 to -0.108) | **<.001** |
|  | -4 | -0.218 (-0.303 to -0.130) | **<.001** |
|  | -3 | -0.211 (-0.296 to -0.122) | **<.001** |
|  | -2 | -0.198 (-0.284 to -0.110) | **<.001** |
|  | -1 | -0.193 (-0.279 to -0.105) | **<.001** |
|  | 1 | -0.184 (-0.270 to -0.095) | **<.001** |
|  | 2 | -0.211 (-0.296 to -0.123) | **<.001** |
|  | 3 | -0.228 (-0.313 to -0.141) | **<.001** |
|  | 4 | -0.258 (-0.340 to -0.171) | **<.001** |
|  | 5 | -0.264 (-0.347 to -0.178) | **<.001** |
|  | 6 | -0.269 (-0.352 to -0.183) | **<.001** |
|  | 7 | -0.273 (-0.355 to -0.187) | **<.001** |
| BSI3 | -7 | -0.089 (-0.178 to 0.003) | .06 |
|  | -6 | -0.049 (-0.140 to 0.042) | .29 |
|  | -5 | -0.069 (-0.159 to 0.021) | .14 |
|  | -4 | -0.071 (-0.161 to 0.020) | .13 |
|  | -3 | -0.087 (-0.176 to 0.004) | .06 |
|  | -2 | -0.098 (-0.187 to -0.008) | **.04** |
|  | -1 | -0.080 (-0.169 to 0.011) | .09 |
|  | 1 | -0.079 (-0.168 to 0.012) | .09 |
|  | 2 | -0.064 (-0.154 to 0.027) | .17 |
|  | 3 | -0.054 (-0.144 to 0.037) | .25 |
|  | 4 | -0.078 (-0.168 to 0.013) | .10 |
|  | 5 | -0.071 (-0.161 to 0.020) | .13 |
|  | 6 | -0.083 (-0.172 to 0.008) | .08 |
|  | 7 | -0.050 (-0.140 to 0.042) | .29 |
| BSI4 | -7 | 0.108 (0.017 to 0.197) | **.02** |
|  | -6 | 0.107 (0.016 to 0.196) | **.02** |
|  | -5 | 0.087 (-0.003 to 0.177) | .06 |
|  | -4 | 0.067 (-0.024 to 0.157) | .15 |
|  | -3 | 0.077 (-0.014 to 0.167) | .10 |
|  | -2 | 0.081 (-0.010 to 0.170) | .08 |
|  | -1 | 0.139 (0.050 to 0.227) | **.003** |
|  | 1 | 0.125 (0.035 to 0.213) | **.007** |
|  | 2 | 0.115 (0.024 to 0.203) | **.01** |
|  | 3 | 0.119 (0.029 to 0.207) | **.01** |
|  | 4 | 0.113 (0.023 to 0.202) | **.02** |
|  | 5 | 0.134 (0.043 to 0.222) | **.004** |
|  | 6 | 0.103 (0.012 to 0.192) | **.03** |
|  | 7 | 0.152 (0.062 to 0.240) | **.001** |
| BSI5 | -7 | 0.188 (0.098 to 0.274) | **<.001** |
|  | -6 | 0.138 (0.047 to 0.226) | **.003** |
|  | -5 | 0.108 (0.017 to 0.197) | **.02** |
|  | -4 | 0.134 (0.044 to 0.222) | **.004** |
|  | -3 | 0.119 (0.029 to 0.207) | **.01** |
|  | -2 | 0.133 (0.043 to 0.221) | **.004** |
|  | -1 | 0.132 (0.042 to 0.220) | **.005** |
|  | 1 | 0.129 (0.039 to 0.217) | **.006** |
|  | 2 | 0.126 (0.035 to 0.214) | **.007** |
|  | 3 | 0.112 (0.021 to 0.200) | **.02** |
|  | 4 | 0.119 (0.028 to 0.207) | **.01** |
|  | 5 | 0.091 (0.000 to 0.181) | .05 |
|  | 6 | 0.095 (0.005 to 0.185) | **.04** |
|  | 7 | 0.132 (0.042 to 0.221) | **.005** |
| BSI6 | -7 | 0.214 (0.126 to 0.299) | **<.001** |
|  | -6 | 0.197 (0.108 to 0.283) | **<.001** |
|  | -5 | 0.192 (0.103 to 0.278) | **<.001** |
|  | -4 | 0.193 (0.104 to 0.279) | **<.001** |
|  | -3 | 0.194 (0.106 to 0.280) | **<.001** |
|  | -2 | 0.212 (0.123 to 0.296) | **<.001** |
|  | -1 | 0.244 (0.157 to 0.327) | **<.001** |
|  | 1 | 0.253 (0.167 to 0.336) | **<.001** |
|  | 2 | 0.226 (0.138 to 0.310) | **<.001** |
|  | 3 | 0.217 (0.129 to 0.302) | **<.001** |
|  | 4 | 0.206 (0.117 to 0.291) | **<.001** |
|  | 5 | 0.218 (0.130 to 0.303) | **<.001** |
|  | 6 | 0.237 (0.149 to 0.321) | **<.001** |
|  | 7 | 0.269 (0.182 to 0.351) | **<.001** |
| BSI7 | -7 | 0.214 (0.125 to 0.299) | **<.001** |
|  | -6 | 0.190 (0.101 to 0.276) | **<.001** |
|  | -5 | 0.144 (0.054 to 0.232) | **.002** |
|  | -4 | 0.151 (0.061 to 0.239) | **.001** |
|  | -3 | 0.163 (0.073 to 0.250) | **<.001** |
|  | -2 | 0.188 (0.099 to 0.274) | **<.001** |
|  | -1 | 0.185 (0.096 to 0.271) | **<.001** |
|  | 1 | 0.238 (0.151 to 0.321) | **<.001** |
|  | 2 | 0.151 (0.062 to 0.239) | **.001** |
|  | 3 | 0.166 (0.077 to 0.253) | **<.001** |
|  | 4 | 0.147 (0.057 to 0.235) | **.002** |
|  | 5 | 0.173 (0.083 to 0.260) | **<.001** |
|  | 6 | 0.166 (0.077 to 0.254) | **<.001** |
|  | 7 | 0.227 (0.139 to 0.312) | **<.001** |
| BSI8 | -7 | 0.129 (0.038 to 0.217) | **.006** |
|  | -6 | 0.094 (0.004 to 0.184) | **.04** |
|  | -5 | 0.084 (-0.007 to 0.173) | .07 |
|  | -4 | 0.074 (-0.017 to 0.164) | .12 |
|  | -3 | 0.094 (0.003 to 0.183) | **.046** |
|  | -2 | 0.097 (0.007 to 0.186) | **.04** |
|  | -1 | 0.119 (0.028 to 0.207) | **.01** |
|  | 1 | 0.120 (0.030 to 0.208) | **.01** |
|  | 2 | 0.100 (0.010 to 0.189) | **.03** |
|  | 3 | 0.065 (-0.026 to 0.155) | .16 |
|  | 4 | 0.064 (-0.027 to 0.154) | .17 |
|  | 5 | 0.062 (-0.029 to 0.152) | .19 |
|  | 6 | 0.078 (-0.013 to 0.168) | .10 |
|  | 7 | 0.104 (0.013 to 0.193) | **.03** |
| BSI9 | -7 | 0.061 (-0.030 to 0.151) | .20 |
|  | -6 | 0.035 (-0.056 to 0.126) | .45 |
|  | -5 | 0.034 (-0.057 to 0.125) | .47 |
|  | -4 | 0.048 (-0.043 to 0.138) | .31 |
|  | -3 | 0.039 (-0.052 to 0.129) | .41 |
|  | -2 | 0.048 (-0.043 to 0.138) | .31 |
|  | -1 | 0.056 (-0.034 to 0.146) | .23 |
|  | 1 | 0.021 (-0.069 to 0.112) | .65 |
|  | 2 | 0.024 (-0.066 to 0.115) | .61 |
|  | 3 | 0.041 (-0.050 to 0.131) | .38 |
|  | 4 | 0.031 (-0.060 to 0.121) | .51 |
|  | 5 | 0.025 (-0.066 to 0.115) | .60 |
|  | 6 | 0.052 (-0.039 to 0.142) | .27 |
|  | 7 | 0.044 (-0.047 to 0.134) | .35 |
| BSI10 | -7 | 0.121 (0.030 to 0.210) | **.01** |
|  | -6 | 0.106 (0.015 to 0.195) | **.02** |
|  | -5 | 0.131 (0.041 to 0.219) | **.005** |
|  | -4 | 0.094 (0.004 to 0.184) | **.04** |
|  | -3 | 0.096 (0.005 to 0.185) | **.04** |
|  | -2 | 0.090 (-0.001 to 0.179) | .06 |
|  | -1 | 0.115 (0.025 to 0.203) | **.01** |
|  | 1 | 0.122 (0.032 to 0.210) | **.01** |
|  | 2 | 0.107 (0.016 to 0.195) | **.02** |
|  | 3 | 0.091 (0.001 to 0.181) | **.05** |
|  | 4 | 0.096 (0.005 to 0.185) | **.04** |
|  | 5 | 0.066 (-0.025 to 0.156) | .16 |
|  | 6 | 0.086 (-0.005 to 0.176) | **.07** |
|  | 7 | 0.105 (0.014 to 0.194) | **.02** |

**Figure 2: Spearman correlation analyses between different surveillance systems and reported cases with a 7-day lag before and after between April 1, 2023 to June 30, 2024**

**
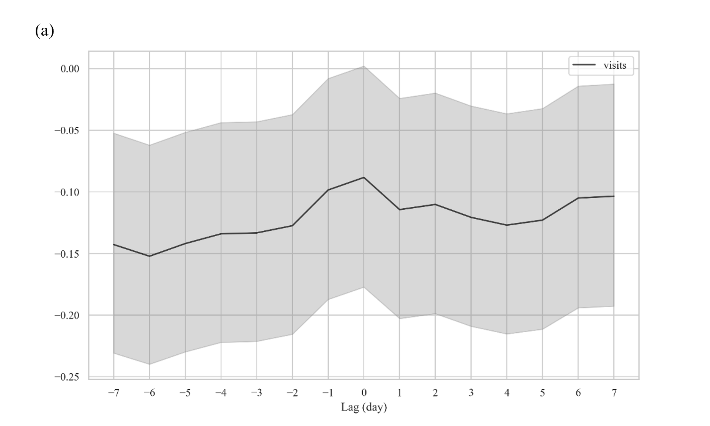

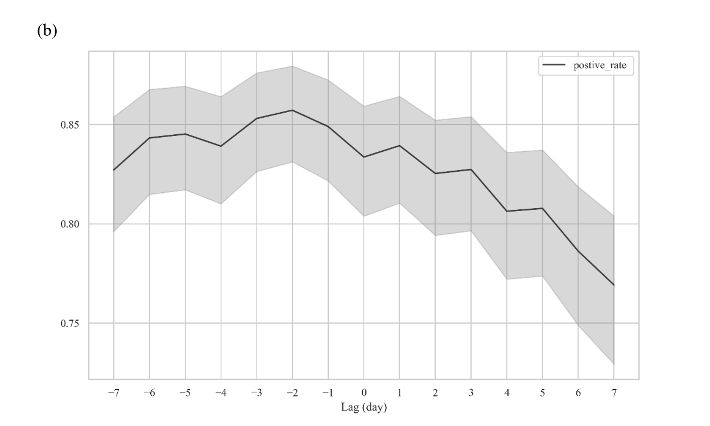
**

**
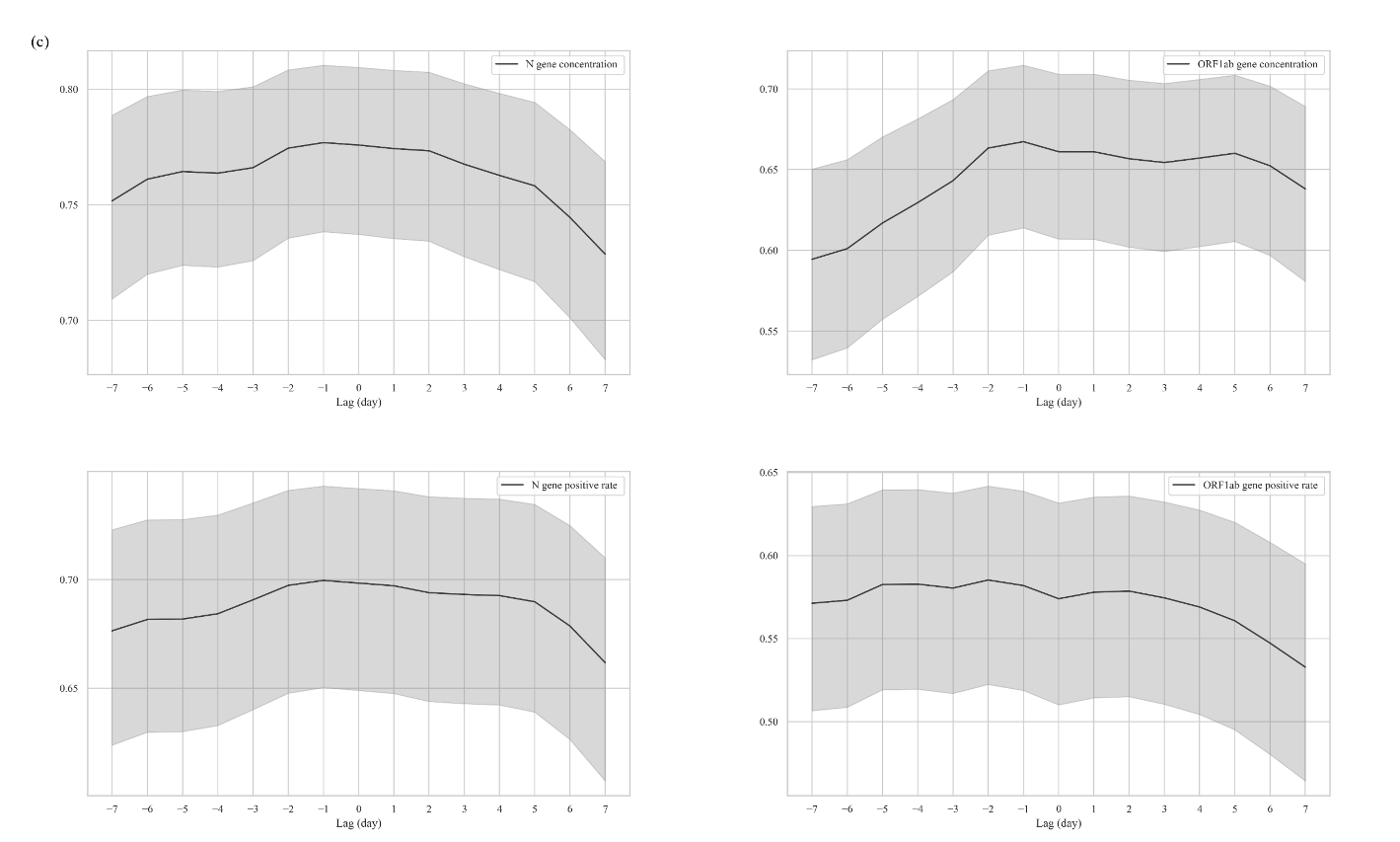
**

**
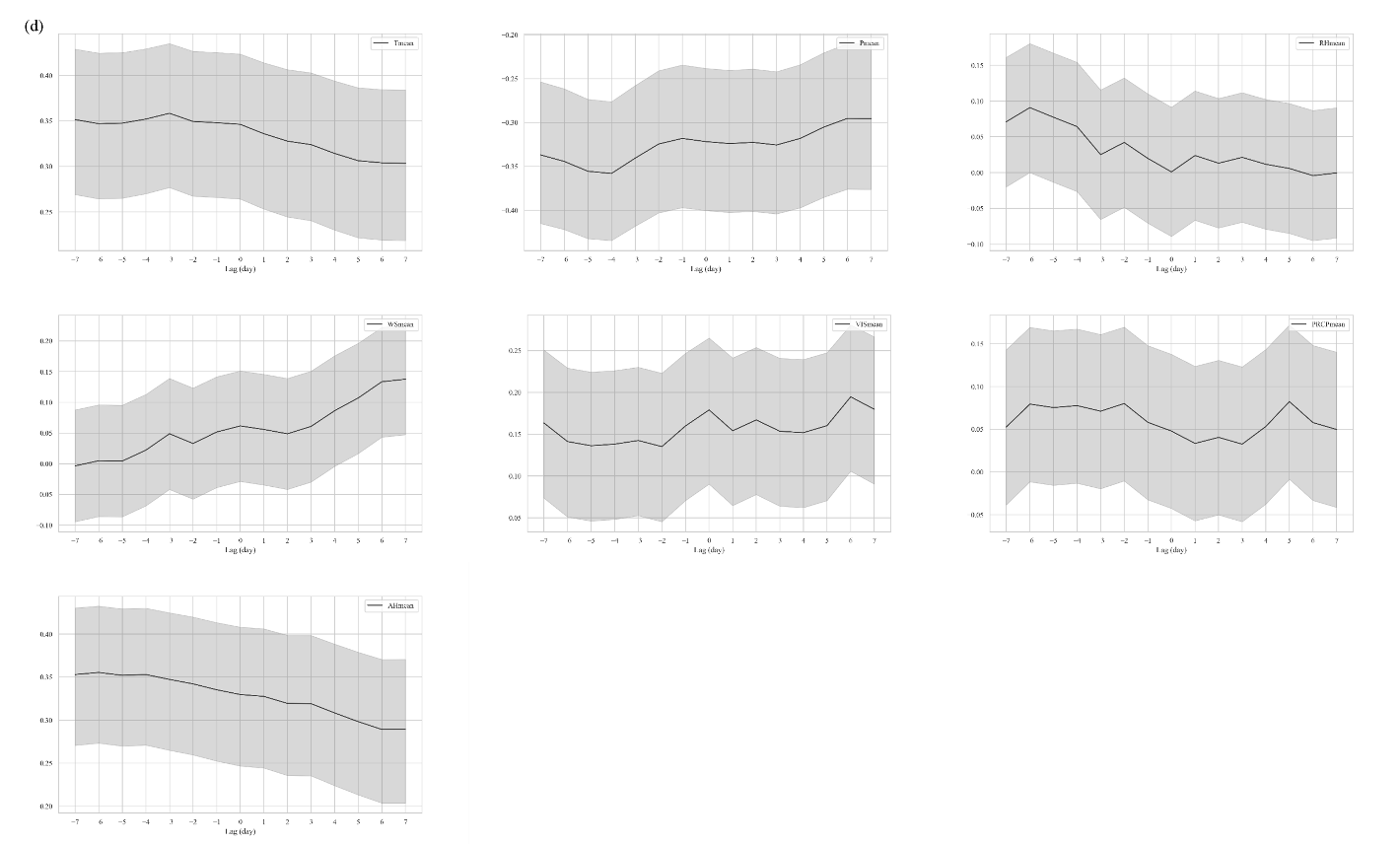
**

**
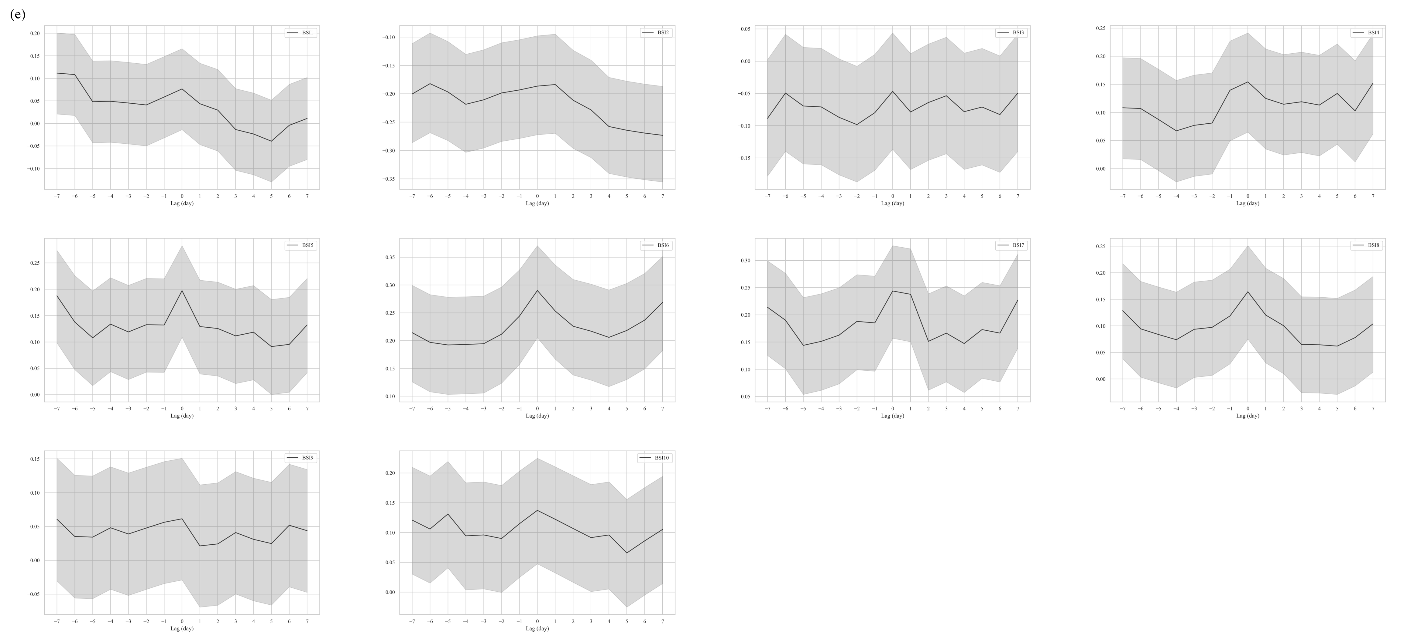
**

(a) visits; (b) positive_rate; (c) wastewater surveillance system; (d) meteorological surveillance system; (e) Internet search engine system.
