# Supplementary material for: Comparative Performance of Wastewater, Clinical, and Digital Surveillance Indicators for COVID-19 Monitoring in Routine Practice: Retrospective Observational Study
Source: J Med Internet Res. 2025 Nov 6;27:e70232. doi: 10.2196/70232 (PMC12592968; doi:10.2196/70232)
Supplement: Multimedia Appendix 5 [file jmir-v27-e70232-s005.docx]

**Table 1. Timeline result between different surveillance systems between April, 2023 to June, 2024**

| Surveillance variables | Model | Lag (day) | Estimate effect | 95% CI | *P* value (uncorrected) | *P* value (corrected) |
| --- | --- | --- | --- | --- | --- | --- |
| **Hospital Surveillance System** |  |  |  |  |  |  |
| positive_rate | With meteorological control | 0 | 0.819 | (0.768 to 0.870) | **<.001** | **<.001** |
|  | With meteorological control | -1 | 0.776 | (0.725 to 0.828) | **<.001** | **<.001** |
|  | With meteorological control | -2 | 0.742 | (0.690 to 0.794) | **<.001** | **<.001** |
|  | With meteorological control | -3 | 0.717 | (0.665 to 0.770) | **<.001** | **<.001** |
|  | With meteorological control | -4 | 0.684 | (0.628 to 0.740) | **<.001** | **<.001** |
|  | With meteorological control | -5 | 0.635 | (0.574 to 0.695) | **<.001** | **<.001** |
|  | With meteorological control | -6 | 0.607 | (0.544 to 0.671) | **<.001** | **<.001** |
|  | With meteorological control | -7 | 0.583 | (0.515 to 0.651) | **<.001** | **<.001** |
|  | Without meteorological control | 0 | 0.780 | (0.731 to 0.828) | **<.001** | **<.001** |
|  | Without meteorological control | -1 | 0.759 | (0.708 to 0.809) | **<.001** | **<.001** |
|  | Without meteorological control | -2 | 0.732 | (0.681 to 0.784) | **<.001** | **<.001** |
|  | Without meteorological control | -3 | 0.716 | (0.664 to 0.768) | **<.001** | **<.001** |
|  | Without meteorological control | -4 | 0.679 | (0.624 to 0.734) | **<.001** | **<.001** |
|  | Without meteorological control | -5 | 0.632 | (0.573 to 0.691) | **<.001** | **<.001** |
|  | Without meteorological control | -6 | 0.608 | (0.545 to 0.671) | **<.001** | **<.001** |
|  | Without meteorological control | -7 | 0.583 | (0.516 to 0.651) | **<.001** | **<.001** |
| **Wastewater Surveillance System** |  |  |  |  |  |  |
| N gene concentration | With meteorological control | 0 | 0.747 | (0.693 to 0.800) | **<.001** | **<.001** |
|  | With meteorological control | -1 | 0.704 | (0.647 to 0.761) | **<.001** | **<.001** |
|  | With meteorological control | -2 | 0.669 | (0.607 to 0.730) | **<.001** | **<.001** |
|  | With meteorological control | -3 | 0.630 | (0.564 to 0.696) | **<.001** | **<.001** |
|  | With meteorological control | -4 | 0.595 | (0.526 to 0.664) | **<.001** | **<.001** |
|  | With meteorological control | -5 | 0.565 | (0.495 to 0.636) | **<.001** | **<.001** |
|  | With meteorological control | -6 | 0.529 | (0.457 to 0.600) | **<.001** | **<.001** |
|  | With meteorological control | -7 | 0.485 | (0.413 to 0.557) | **<.001** | **<.001** |
|  | Without meteorological control | 0 | 0.745 | (0.692 to 0.798) | **<.001** | **<.001** |
|  | Without meteorological control | -1 | 0.710 | (0.653 to 0.766) | **<.001** | **<.001** |
|  | Without meteorological control | -2 | 0.673 | (0.613 to 0.733) | **<.001** | **<.001** |
|  | Without meteorological control | -3 | 0.630 | (0.567 to 0.694) | **<.001** | **<.001** |
|  | Without meteorological control | -4 | 0.592 | (0.525 to 0.659) | **<.001** | **<.001** |
|  | Without meteorological control | -5 | 0.566 | (0.496 to 0.635) | **<.001** | **<.001** |
|  | Without meteorological control | -6 | 0.534 | (0.463 to 0.606) | **<.001** | **<.001** |
|  | Without meteorological control | -7 | 0.498 | (0.425 to 0.570) | **<.001** | **<.001** |
| N gene positive rate | With meteorological control | 0 | 1.023 | (0.931 to 1.115) | **<.001** | **<.001** |
|  | With meteorological control | -1 | 0.999 | (0.906 to 1.092) | **<.001** | **<.001** |
|  | With meteorological control | -2 | 0.991 | (0.898 to 1.085) | **<.001** | **<.001** |
|  | With meteorological control | -3 | 0.971 | (0.876 to 1.067) | **<.001** | **<.001** |
|  | With meteorological control | -4 | 0.946 | (0.850 to 1.042) | **<.001** | **<.001** |
|  | With meteorological control | -5 | 0.935 | (0.840 to 1.029) | **<.001** | **<.001** |
|  | With meteorological control | -6 | 0.909 | (0.814 to 1.003) | **<.001** | **<.001** |
|  | With meteorological control | -7 | 0.876 | (0.781 to 0.970) | **<.001** | **<.001** |
|  | Without meteorological control | 0 | 1.017 | (0.927 to 1.107) | **<.001** | **<.001** |
|  | Without meteorological control | -1 | 1.007 | (0.916 to 1.098) | **<.001** | **<.001** |
|  | Without meteorological control | -2 | 1.009 | (0.917 to 1.101) | **<.001** | **<.001** |
|  | Without meteorological control | -3 | 0.993 | (0.899 to 1.087) | **<.001** | **<.001** |
|  | Without meteorological control | -4 | 0.968 | (0.872 to 1.063) | **<.001** | **<.001** |
|  | Without meteorological control | -5 | 0.959 | (0.863 to 1.054) | **<.001** | **<.001** |
|  | Without meteorological control | -6 | 0.934 | (0.837 to 1.030) | **<.001** | **<.001** |
|  | Without meteorological control | -7 | 0.898 | (0.801 to 0.995) | **<.001** | **<.001** |
| ORF1ab gene concentration | With meteorological control | 0 | 0.660 | (0.610 to 0.709) | **<.001** | **<.001** |
|  | With meteorological control | -1 | 0.633 | (0.581 to 0.684) | **<.001** | **<.001** |
|  | With meteorological control | -2 | 0.604 | (0.550 to 0.659) | **<.001** | **<.001** |
|  | With meteorological control | -3 | 0.576 | (0.519 to 0.633) | **<.001** | **<.001** |
|  | With meteorological control | -4 | 0.548 | (0.488 to 0.608) | **<.001** | **<.001** |
|  | With meteorological control | -5 | 0.520 | (0.458 to 0.582) | **<.001** | **<.001** |
|  | With meteorological control | -6 | 0.485 | (0.421 to 0.549) | **<.001** | **<.001** |
|  | With meteorological control | -7 | 0.441 | (0.376 to 0.507) | **<.001** | **<.001** |
|  | Without meteorological control | 0 | 0.640 | (0.593 to 0.686) | **<.001** | **<.001** |
|  | Without meteorological control | -1 | 0.619 | (0.570 to 0.668) | **<.001** | **<.001** |
|  | Without meteorological control | -2 | 0.593 | (0.541 to 0.645) | **<.001** | **<.001** |
|  | Without meteorological control | -3 | 0.564 | (0.508 to 0.619) | **<.001** | **<.001** |
|  | Without meteorological control | -4 | 0.533 | (0.475 to 0.591) | **<.001** | **<.001** |
|  | Without meteorological control | -5 | 0.511 | (0.450 to 0.572) | **<.001** | **<.001** |
|  | Without meteorological control | -6 | 0.485 | (0.422 to 0.548) | **<.001** | **<.001** |
|  | Without meteorological control | -7 | 0.453 | (0.388 to 0.518) | **<.001** | **<.001** |
| ORF1ab gene positive rate | With meteorological control | 0 | 0.836 | (0.780 to 0.893) | **<.001** | **<.001** |
|  | With meteorological control | -1 | 0.806 | (0.747 to 0.865) | **<.001** | **<.001** |
|  | With meteorological control | -2 | 0.771 | (0.709 to 0.833) | **<.001** | **<.001** |
|  | With meteorological control | -3 | 0.729 | (0.663 to 0.796) | **<.001** | **<.001** |
|  | With meteorological control | -4 | 0.690 | (0.620 to 0.761) | **<.001** | **<.001** |
|  | With meteorological control | -5 | 0.661 | (0.587 to 0.734) | **<.001** | **<.001** |
|  | With meteorological control | -6 | 0.626 | (0.550 to 0.702) | **<.001** | **<.001** |
|  | With meteorological control | -7 | 0.583 | (0.505 to 0.661) | **<.001** | **<.001** |
|  | Without meteorological control | 0 | 0.809 | (0.754 to 0.864) | **<.001** | **<.001** |
|  | Without meteorological control | -1 | 0.785 | (0.728 to 0.843) | **<.001** | **<.001** |
|  | Without meteorological control | -2 | 0.758 | (0.697 to 0.819) | **<.001** | **<.001** |
|  | Without meteorological control | -3 | 0.724 | (0.659 to 0.788) | **<.001** | **<.001** |
|  | Without meteorological control | -4 | 0.690 | (0.622 to 0.759) | **<.001** | **<.001** |
|  | Without meteorological control | -5 | 0.663 | (0.592 to 0.734) | **<.001** | **<.001** |
|  | Without meteorological control | -6 | 0.631 | (0.557 to 0.705) | **<.001** | **<.001** |
|  | Without meteorological control | -7 | 0.592 | (0.516 to 0.668) | **<.001** | **<.001** |
| **Internet Search Engine System** |  |  |  |  |  |  |
| BSI2, Baidu search rank for “cough” | With meteorological control | 0 | 0.667 | (0.507 to 0.828) | **<.001** | **<.001** |
|  | With meteorological control | -1 | 0.560 | (0.405 to 0.715) | **<.001** | **<.001** |
|  | With meteorological control | -2 | 0.536 | (0.374 to 0.698) | **<.001** | **<.001** |
|  | With meteorological control | -3 | 0.472 | (0.316 to 0.628) | **<.001** | **<.001** |
|  | With meteorological control | -4 | 0.356 | (0.199 to 0.513) | **<.001** | **<.001** |
|  | With meteorological control | -5 | 0.241 | (0.085 to 0.397) | **<.001** | **.003** |
|  | With meteorological control | -6 | 0.186 | (0.032 to 0.340) | **.02** | **.02** |
|  | With meteorological control | -7 | 0.147 | (0.000 to 0.293) | .05 | .06 |
|  | Without meteorological control | 0 | 0.693 | (0.536 to 0.850) | **<.001** | **<.001** |
|  | Without meteorological control | -1 | 0.584 | (0.428 to 0.741) | **<.001** | **<.001** |
|  | Without meteorological control | -2 | 0.546 | (0.386 to 0.707) | **<.001** | **<.001** |
|  | Without meteorological control | -3 | 0.483 | (0.326 to 0.640) | **<.001** | **<.001** |
|  | Without meteorological control | -4 | 0.369 | (0.214 to 0.524) | **<.001** | **<.001** |
|  | Without meteorological control | -5 | 0.272 | (0.117 to 0.427) | **<.001** | **.001** |
|  | Without meteorological control | -6 | 0.223 | (0.071 to 0.375) | **<.001** | **.005** |
|  | Without meteorological control | -7 | 0.174 | (0.025 to 0.322) | **.02** | **.03** |
| BSI4, Baidu search rank for “weakness” | With meteorological control | 0 | -0.004 | (-0.097 to 0.090) | .94 | .95 |
|  | With meteorological control | -1 | -0.039 | (-0.134 to 0.056) | .42 | .47 |
|  | With meteorological control | -2 | -0.056 | (-0.149 to 0.038) | .25 | .29 |
|  | With meteorological control | -3 | -0.027 | (-0.119 to 0.066) | .57 | .62 |
|  | With meteorological control | -4 | -0.018 | (-0.113 to 0.076) | .70 | .75 |
|  | With meteorological control | -5 | -0.036 | (-0.131 to 0.059) | .46 | .51 |
|  | With meteorological control | -6 | -0.012 | (-0.107 to 0.083) | .80 | .84 |
|  | With meteorological control | -7 | 0.001 | (-0.092 to 0.093) | .99 | .99 |
|  | Without meteorological control | 0 | 0.010 | (-0.084 to 0.104) | .83 | .86 |
|  | Without meteorological control | -1 | -0.034 | (-0.129 to 0.062) | .49 | .54 |
|  | Without meteorological control | -2 | -0.056 | (-0.151 to 0.039) | .25 | .29 |
|  | Without meteorological control | -3 | -0.042 | (-0.136 to 0.053) | .39 | .44 |
|  | Without meteorological control | -4 | -0.026 | (-0.122 to 0.070) | .60 | .64 |
|  | Without meteorological control | -5 | -0.029 | (-0.124 to 0.067) | .55 | .60 |
|  | Without meteorological control | -6 | 0.003 | (-0.092 to 0.099) | .94 | .95 |
|  | Without meteorological control | -7 | -0.017 | (-0.110 to 0.076) | .72 | .76 |
| BSI5, Baidu search rank for “diarrhea” | With meteorological control | 0 | 0.116 | (0.018 to 0.214) | **.02** | **.03** |
|  | With meteorological control | -1 | 0.052 | (-0.040 to 0.144) | .27 | .31 |
|  | With meteorological control | -2 | 0.029 | (-0.060 to 0.118) | .52 | .56 |
|  | With meteorological control | -3 | 0.029 | (-0.058 to 0.117) | .51 | .56 |
|  | With meteorological control | -4 | 0.041 | (-0.047 to 0.129) | .36 | .41 |
|  | With meteorological control | -5 | 0.010 | (-0.075 to 0.096) | .81 | .84 |
|  | With meteorological control | -6 | 0.003 | (-0.086 to 0.091) | .95 | .96 |
|  | With meteorological control | -7 | -0.012 | (-0.102 to 0.078) | .79 | .83 |
|  | Without meteorological control | 0 | 0.085 | (-0.012 to 0.182) | .09 | .10 |
|  | Without meteorological control | -1 | 0.041 | (-0.050 to 0.131) | .38 | .43 |
|  | Without meteorological control | -2 | 0.039 | (-0.050 to 0.128) | .39 | .44 |
|  | Without meteorological control | -3 | 0.036 | (-0.051 to 0.124) | .42 | .47 |
|  | Without meteorological control | -4 | 0.049 | (-0.040 to 0.137) | .28 | .32 |
|  | Without meteorological control | -5 | 0.008 | (-0.079 to 0.095) | .85 | .87 |
|  | Without meteorological control | -6 | -0.007 | (-0.095 to 0.081) | .87 | .89 |
|  | Without meteorological control | -7 | -0.043 | (-0.131 to 0.046) | .34 | .39 |
| BSI6, Baidu search rank for “COVID-19” | With meteorological control | 0 | 0.469 | (0.417 to 0.520) | **<.001** | **<.001** |
|  | With meteorological control | -1 | 0.433 | (0.380 to 0.486) | **<.001** | **<.001** |
|  | With meteorological control | -2 | 0.413 | (0.358 to 0.468) | **<.001** | **<.001** |
|  | With meteorological control | -3 | 0.416 | (0.357 to 0.475) | **<.001** | **<.001** |
|  | With meteorological control | -4 | 0.398 | (0.338 to 0.458) | **<.001** | **<.001** |
|  | With meteorological control | -5 | 0.386 | (0.328 to 0.444) | **<.001** | **<.001** |
|  | With meteorological control | -6 | 0.409 | (0.346 to 0.472) | **<.001** | **<.001** |
|  | With meteorological control | -7 | 0.398 | (0.339 to 0.457) | **<.001** | **<.001** |
|  | Without meteorological control | 0 | 0.478 | (0.429 to 0.527) | **<.001** | **<.001** |
|  | Without meteorological control | -1 | 0.419 | (0.371 to 0.467) | **<.001** | **<.001** |
|  | Without meteorological control | -2 | 0.405 | (0.356 to 0.455) | **<.001** | **<.001** |
|  | Without meteorological control | -3 | 0.402 | (0.349 to 0.456) | **<.001** | **<.001** |
|  | Without meteorological control | -4 | 0.399 | (0.345 to 0.453) | **<.001** | **<.001** |
|  | Without meteorological control | -5 | 0.381 | (0.330 to 0.432) | **<.001** | **<.001** |
|  | Without meteorological control | -6 | 0.395 | (0.342 to 0.448) | **<.001** | **<.001** |
|  | Without meteorological control | -7 | 0.407 | (0.354 to 0.460) | **<.001** | **<.001** |
| BSI7, Baidu search rank for “novel coronavirus” | With meteorological control | 0 | 0.540 | (0.410 to 0.671) | **<.001** | **<.001** |
|  | With meteorological control | -1 | 0.470 | (0.347 to 0.592) | **<.001** | **<.001** |
|  | With meteorological control | -2 | 0.333 | (0.215 to 0.452) | **<.001** | **<.001** |
|  | With meteorological control | -3 | 0.359 | (0.242 to 0.477) | **<.001** | **<.001** |
|  | With meteorological control | -4 | 0.291 | (0.169 to 0.412) | **<.001** | **<.001** |
|  | With meteorological control | -5 | 0.323 | (0.203 to 0.443) | **<.001** | **<.001** |
|  | With meteorological control | -6 | 0.32 | (0.199 to 0.441) | **<.001** | **<.001** |
|  | With meteorological control | -7 | 0.346 | (0.220 to 0.472) | **<.001** | **<.001** |
|  | Without meteorological control | 0 | 0.589 | (0.458 to 0.719) | **<.001** | **<.001** |
|  | Without meteorological control | -1 | 0.511 | (0.387 to 0.636) | **<.001** | **<.001** |
|  | Without meteorological control | -2 | 0.381 | (0.263 to 0.500) | **<.001** | **<.001** |
|  | Without meteorological control | -3 | 0.399 | (0.281 to 0.518) | **<.001** | **<.001** |
|  | Without meteorological control | -4 | 0.355 | (0.235 to 0.475) | **<.001** | **<.001** |
|  | Without meteorological control | -5 | 0.383 | (0.265 to 0.501) | **<.001** | **<.001** |
|  | Without meteorological control | -6 | 0.386 | (0.265 to 0.507) | **<.001** | **<.001** |
|  | Without meteorological control | -7 | 0.403 | (0.275 to 0.532) | **<.001** | **<.001** |
| BSI8, Baidu search rank for “COVID-19 pneumonia” | With meteorological control | 0 | 0.576 | (0.443 to 0.708) | **<.001** | **<.001** |
|  | With meteorological control | -1 | 0.464 | (0.340 to 0.587) | **<.001** | **<.001** |
|  | With meteorological control | -2 | 0.386 | (0.264 to 0.508) | **<.001** | **<.001** |
|  | With meteorological control | -3 | 0.333 | (0.215 to 0.451) | **<.001** | **<.001** |
|  | With meteorological control | -4 | 0.352 | (0.234 to 0.469) | **<.001** | **<.001** |
|  | With meteorological control | -5 | 0.288 | (0.169 to 0.407) | **<.001** | **<.001** |
|  | With meteorological control | -6 | 0.264 | (0.148 to 0.379) | **<.001** | **<.001** |
|  | With meteorological control | -7 | 0.312 | (0.184 to 0.439) | **<.001** | **<.001** |
|  | Without meteorological control | 0 | 0.618 | (0.481 to 0.755) | **<.001** | **<.001** |
|  | Without meteorological control | -1 | 0.497 | (0.372 to 0.621) | **<.001** | **<.001** |
|  | Without meteorological control | -2 | 0.424 | (0.303 to 0.545) | **<.001** | **<.001** |
|  | Without meteorological control | -3 | 0.372 | (0.256 to 0.489) | **<.001** | **<.001** |
|  | Without meteorological control | -4 | 0.406 | (0.290 to 0.523) | **<.001** | **<.001** |
|  | Without meteorological control | -5 | 0.347 | (0.230 to 0.464) | **<.001** | **<.001** |
|  | Without meteorological control | -6 | 0.320 | (0.204 to 0.437) | **<.001** | **<.001** |
|  | Without meteorological control | -7 | 0.369 | (0.241 to 0.497) | **<.001** | **<.001** |
| BSI10, Baidu search rank for “Omicron” | With meteorological control | 0 | 0.193 | (0.086 to 0.299) | **<.001** | **<.001** |
|  | With meteorological control | -1 | 0.203 | (0.098 to 0.307) | **<.001** | **<.001** |
|  | With meteorological control | -2 | 0.215 | (0.110 to 0.321) | **<.001** | **<.001** |
|  | With meteorological control | -3 | 0.216 | (0.112 to 0.320) | **<.001** | **<.001** |
|  | With meteorological control | -4 | 0.215 | (0.111 to 0.319) | **<.001** | **<.001** |
|  | With meteorological control | -5 | 0.156 | (0.054 to 0.259) | **<.001** | **<.001** |
|  | With meteorological control | -6 | 0.153 | (0.049 to 0.257) | **<.001** | **.01** |
|  | With meteorological control | -7 | 0.183 | (0.078 to 0.288) | **<.001** | **<.001** |
|  | Without meteorological control | 0 | 0.224 | (0.118 to 0.330) | **<.001** | **<.001** |
|  | Without meteorological control | -1 | 0.234 | (0.130 to 0.337) | **<.001** | **<.001** |
|  | Without meteorological control | -2 | 0.223 | (0.119 to 0.327) | **<.001** | **<.001** |
|  | Without meteorological control | -3 | 0.217 | (0.113 to 0.321) | **<.001** | **<.001** |
|  | Without meteorological control | -4 | 0.232 | (0.127 to 0.337) | **<.001** | **<.001** |
|  | Without meteorological control | -5 | 0.178 | (0.074 to 0.281) | **<.001** | **<.001** |
|  | Without meteorological control | -6 | 0.153 | (0.048 to 0.257) | **<.001** | **.01** |
|  | Without meteorological control | -7 | 0.184 | (0.079 to 0.290) | **<.001** | **<.001** |

**Table 2. Interaction result between different surveillance systems and meteorological variables between April, 2023 to June, 2024**

| Lag | Surveillance Variable | Meteorological Variable | Interaction Coefficient | SE | *P* value | RR (Interaction) | RR 95%CI | Main Effect (Surveillance) | Main Effect (Meteorological) |
| --- | --- | --- | --- | --- | --- | --- | --- | --- | --- |
| 0 | postive_rate | Tmean | -0.063 | 0.040 | 0.117 | 0.939 | (0.868 to 1.016) | 0.796 | 0.033 |
| 0 | postive_rate | Pmean | 0.033 | 0.035 | 0.347 | 1.034 | (0.965 to 1.107) | 0.797 | -0.086 |
| 0 | postive_rate | AHmean | -0.130 | 0.035 | <0.001 | 0.878 | (0.819 to 0.941) | 0.802 | 0.013 |
| 0 | postive_rate | VISmean | 0.142 | 0.027 | <0.001 | 1.152 | (1.094 to 1.214) | 0.836 | 0.018 |
| 0 | N_gene_positive_rate | Tmean | -0.058 | 0.073 | 0.431 | 0.944 | (0.819 to 1.089) | 1.025 | -0.033 |
| 0 | N_gene_positive_rate | Pmean | -0.031 | 0.064 | 0.622 | 0.969 | (0.855 to 1.098) | 1.015 | -0.017 |
| 0 | N_gene_positive_rate | AHmean | -0.155 | 0.066 | 0.019 | 0.856 | (0.753 to 0.974) | 1.044 | 0.199 |
| 0 | N_gene_positive_rate | VISmean | 0.001 | 0.047 | 0.979 | 1.001 | (0.913 to 1.098) | 1.018 | -0.147 |
| 0 | N_gene_concentration | Tmean | -0.056 | 0.047 | 0.232 | 0.945 | (0.862 to 1.036) | 0.758 | -0.098 |
| 0 | N_gene_concentration | Pmean | 0.043 | 0.040 | 0.283 | 1.044 | (0.965 to 1.129) | 0.763 | -0.057 |
| 0 | N_gene_concentration | AHmean | -0.078 | 0.042 | 0.061 | 0.925 | (0.853 to 1.004) | 0.754 | 0.017 |
| 0 | N_gene_concentration | VISmean | 0.028 | 0.031 | 0.369 | 1.028 | (0.968 to 1.091) | 0.742 | -0.056 |
| 0 | ORF1ab_gene_positive_rate | Tmean | -0.153 | 0.041 | <0.001 | 0.858 | (0.791 to 0.930) | 0.877 | 0.173 |
| 0 | ORF1ab_gene_positive_rate | Pmean | 0.011 | 0.031 | 0.725 | 1.011 | (0.951 to 1.075) | 0.835 | -0.166 |
| 0 | ORF1ab_gene_positive_rate | AHmean | -0.155 | 0.034 | <0.001 | 0.856 | (0.801 to 0.916) | 0.874 | 0.300 |
| 0 | ORF1ab_gene_positive_rate | VISmean | 0.012 | 0.025 | 0.624 | 1.012 | (0.964 to 1.062) | 0.810 | -0.007 |
| 0 | ORF1ab_gene_concentration | Tmean | -0.061 | 0.044 | 0.166 | 0.941 | (0.863 to 1.026) | 0.666 | 0.116 |
| 0 | ORF1ab_gene_concentration | Pmean | 0.024 | 0.035 | 0.490 | 1.025 | (0.957 to 1.097) | 0.657 | -0.102 |
| 0 | ORF1ab_gene_concentration | AHmean | -0.09 | 0.039 | 0.020 | 0.914 | (0.848 to 0.986) | 0.665 | 0.122 |
| 0 | ORF1ab_gene_concentration | VISmean | 0.076 | 0.027 | 0.005 | 1.079 | (1.024 to 1.138) | 0.657 | 0.016 |
| 0 | BSI2 | Tmean | 0.194 | 0.099 | 0.050 | 1.214 | (1.001 to 1.474) | 0.667 | -0.344 |
| 0 | BSI2 | Pmean | -0.047 | 0.106 | 0.659 | 0.954 | (0.775 to 1.175) | 0.685 | 0.262 |
| 0 | BSI2 | AHmean | 0.173 | 0.092 | 0.061 | 1.188 | (0.992 to 1.423) | 0.703 | -0.382 |
| 0 | BSI2 | VISmean | 0.112 | 0.077 | 0.148 | 1.119 | (0.961 to 1.302) | 0.697 | -0.025 |
| 0 | BSI4 | Tmean | 0.068 | 0.062 | 0.274 | 1.071 | (0.948 to 1.210) | -0.012 | -0.357 |
| 0 | BSI4 | Pmean | -0.069 | 0.062 | 0.264 | 0.933 | (0.827 to 1.053) | -0.016 | 0.248 |
| 0 | BSI4 | AHmean | 0.056 | 0.058 | 0.335 | 1.057 | (0.944 to 1.183) | -0.019 | -0.371 |
| 0 | BSI4 | VISmean | -0.045 | 0.056 | 0.421 | 0.956 | (0.857 to 1.067) | 0.028 | -0.162 |
| 0 | BSI5 | Tmean | -0.311 | 0.066 | <0.001 | 0.733 | (0.644 to 0.834) | 0.176 | -0.396 |
| 0 | BSI5 | Pmean | 0.269 | 0.068 | <0.001 | 1.309 | (1.146 to 1.494) | 0.176 | 0.220 |
| 0 | BSI5 | AHmean | -0.231 | 0.058 | <0.001 | 0.794 | (0.709 to 0.889) | 0.124 | -0.338 |
| 0 | BSI5 | VISmean | 0.081 | 0.056 | 0.149 | 1.084 | (0.972 to 1.210) | 0.100 | -0.199 |
| 0 | BSI6 | Tmean | 0.371 | 0.038 | <0.001 | 1.449 | (1.345 to 1.561) | 0.405 | -0.410 |
| 0 | BSI6 | Pmean | -0.213 | 0.026 | <0.001 | 0.808 | (0.768 to 0.850) | 0.456 | 0.356 |
| 0 | BSI6 | AHmean | 0.338 | 0.036 | <0.001 | 1.402 | (1.306 to 1.505) | 0.455 | -0.522 |
| 0 | BSI6 | VISmean | 0.094 | 0.028 | <0.001 | 1.099 | (1.039 to 1.162) | 0.481 | -0.140 |
| 0 | BSI7 | Tmean | 0.287 | 0.066 | <0.001 | 1.333 | (1.170 to 1.518) | 0.536 | -0.397 |
| 0 | BSI7 | Pmean | -0.313 | 0.058 | <0.001 | 0.732 | (0.652 to 0.82) | 0.503 | 0.374 |
| 0 | BSI7 | AHmean | 0.360 | 0.072 | <0.001 | 1.434 | (1.245 to 1.652) | 0.552 | -0.467 |
| 0 | BSI7 | VISmean | 0.100 | 0.072 | 0.165 | 1.105 | (0.960 to 1.271) | 0.578 | -0.158 |
| 0 | BSI8 | Tmean | 0.315 | 0.087 | <0.001 | 1.371 | (1.156 to 1.626) | 0.516 | -0.476 |
| 0 | BSI8 | Pmean | -0.241 | 0.077 | 0.002 | 0.786 | (0.676 to 0.913) | 0.506 | 0.315 |
| 0 | BSI8 | AHmean | 0.275 | 0.086 | 0.002 | 1.317 | (1.112 to 1.560) | 0.543 | -0.453 |
| 0 | BSI8 | VISmean | 0.214 | 0.081 | 0.008 | 1.239 | (1.057 to 1.451) | 0.653 | -0.243 |
| 0 | BSI10 | Tmean | 0.025 | 0.066 | 0.711 | 1.025 | (0.900 to 1.167) | 0.210 | -0.332 |
| 0 | BSI10 | Pmean | -0.037 | 0.066 | 0.578 | 0.964 | (0.847 to 1.097) | 0.201 | 0.224 |
| 0 | BSI10 | AHmean | 0.009 | 0.062 | 0.887 | 1.009 | (0.894 to 1.139) | 0.204 | -0.310 |
| 0 | BSI10 | VISmean | 0.091 | 0.053 | 0.083 | 1.096 | (0.988 to 1.214) | 0.224 | -0.211 |
| 1 | postive_rate | Tmean | -0.048 | 0.040 | 0.230 | 0.953 | (0.881 to 1.031) | 0.768 | -0.035 |
| 1 | postive_rate | Pmean | 0.035 | 0.037 | 0.348 | 1.036 | (0.963 to 1.114) | 0.777 | -0.082 |
| 1 | postive_rate | AHmean | -0.100 | 0.036 | 0.006 | 0.905 | (0.843 to 0.972) | 0.771 | -0.008 |
| 1 | postive_rate | VISmean | 0.084 | 0.028 | 0.002 | 1.088 | (1.031 to 1.148) | 0.780 | -0.007 |
| 1 | N_gene_positive_rate | Tmean | -0.079 | 0.074 | 0.286 | 0.924 | (0.799 to 1.068) | 1.018 | -0.057 |
| 1 | N_gene_positive_rate | Pmean | 0.008 | 0.066 | 0.905 | 1.008 | (0.886 to 1.147) | 1.008 | 0.003 |
| 1 | N_gene_positive_rate | AHmean | -0.180 | 0.068 | 0.008 | 0.835 | (0.732 to 0.954) | 1.027 | 0.147 |
| 1 | N_gene_positive_rate | VISmean | -0.005 | 0.048 | 0.917 | 0.995 | (0.905 to 1.094) | 1.004 | -0.112 |
| 1 | N_gene_concentration | Tmean | -0.075 | 0.052 | 0.149 | 0.928 | (0.838 to 1.027) | 0.729 | -0.104 |
| 1 | N_gene_concentration | Pmean | 0.108 | 0.044 | 0.014 | 1.114 | (1.023 to 1.214) | 0.755 | -0.061 |
| 1 | N_gene_concentration | AHmean | -0.116 | 0.046 | 0.011 | 0.890 | (0.814 to 0.974) | 0.726 | -0.013 |
| 1 | N_gene_concentration | VISmean | 0.020 | 0.034 | 0.555 | 1.020 | (0.955 to 1.090) | 0.708 | -0.031 |
| 1 | ORF1ab_gene_positive_rate | Tmean | -0.184 | 0.044 | <0.001 | 0.832 | (0.763 to 0.907) | 0.865 | 0.144 |
| 1 | ORF1ab_gene_positive_rate | Pmean | 0.048 | 0.034 | 0.159 | 1.050 | (0.981 to 1.123) | 0.823 | -0.168 |
| 1 | ORF1ab_gene_positive_rate | AHmean | -0.188 | 0.037 | <0.001 | 0.829 | (0.770 to 0.891) | 0.859 | 0.272 |
| 1 | ORF1ab_gene_positive_rate | VISmean | 0.008 | 0.027 | 0.769 | 1.008 | (0.957 to 1.062) | 0.786 | -0.002 |
| 1 | ORF1ab_gene_concentration | Tmean | -0.098 | 0.048 | 0.044 | 0.907 | (0.825 to 0.997) | 0.657 | 0.083 |
| 1 | ORF1ab_gene_concentration | Pmean | 0.088 | 0.038 | 0.021 | 1.093 | (1.014 to 1.178) | 0.664 | -0.103 |
| 1 | ORF1ab_gene_concentration | AHmean | -0.138 | 0.041 | <0.001 | 0.871 | (0.804 to 0.944) | 0.658 | 0.072 |
| 1 | ORF1ab_gene_concentration | VISmean | 0.070 | 0.030 | 0.019 | 1.072 | (1.012 to 1.136) | 0.641 | 0.045 |
| 1 | BSI2 | Tmean | 0.239 | 0.097 | 0.014 | 1.271 | (1.050 to 1.537) | 0.566 | -0.379 |
| 1 | BSI2 | Pmean | -0.182 | 0.103 | 0.078 | 0.834 | (0.681 to 1.020) | 0.544 | 0.235 |
| 1 | BSI2 | AHmean | 0.232 | 0.088 | 0.008 | 1.261 | (1.063 to 1.497) | 0.600 | -0.362 |
| 1 | BSI2 | VISmean | 0.115 | 0.077 | 0.135 | 1.121 | (0.965 to 1.303) | 0.571 | -0.061 |
| 1 | BSI4 | Tmean | 0.033 | 0.063 | 0.605 | 1.033 | (0.913 to 1.169) | -0.050 | -0.356 |
| 1 | BSI4 | Pmean | -0.078 | 0.062 | 0.212 | 0.925 | (0.820 to 1.045) | -0.060 | 0.253 |
| 1 | BSI4 | AHmean | 0.087 | 0.06 | 0.146 | 1.091 | (0.970 to 1.226) | -0.053 | -0.365 |
| 1 | BSI4 | VISmean | -0.149 | 0.054 | 0.006 | 0.862 | (0.775 to 0.959) | -0.018 | -0.153 |
| 1 | BSI5 | Tmean | -0.210 | 0.062 | <0.001 | 0.810 | (0.718 to 0.915) | 0.103 | -0.379 |
| 1 | BSI5 | Pmean | 0.288 | 0.062 | <0.001 | 1.334 | (1.182 to 1.505) | 0.121 | 0.243 |
| 1 | BSI5 | AHmean | -0.215 | 0.056 | <0.001 | 0.806 | (0.722 to 0.900) | 0.063 | -0.347 |
| 1 | BSI5 | VISmean | 0.071 | 0.05 | 0.156 | 1.074 | (0.973 to 1.185) | 0.047 | -0.186 |
| 1 | BSI6 | Tmean | 0.428 | 0.039 | <0.001 | 1.534 | (1.42 to 1.656) | 0.399 | -0.321 |
| 1 | BSI6 | Pmean | -0.274 | 0.027 | <0.001 | 0.761 | (0.721 to 0.802) | 0.434 | 0.282 |
| 1 | BSI6 | AHmean | 0.338 | 0.035 | <0.001 | 1.402 | (1.310 to 1.501) | 0.421 | -0.449 |
| 1 | BSI6 | VISmean | 0.034 | 0.025 | 0.184 | 1.034 | (0.984 to 1.086) | 0.413 | -0.115 |
| 1 | BSI7 | Tmean | 0.230 | 0.071 | 0.001 | 1.259 | (1.095 to 1.448) | 0.472 | -0.336 |
| 1 | BSI7 | Pmean | -0.232 | 0.069 | <0.001 | 0.793 | (0.693 to 0.907) | 0.446 | 0.264 |
| 1 | BSI7 | AHmean | 0.274 | 0.070 | <0.001 | 1.315 | (1.147 to 1.508) | 0.472 | -0.393 |
| 1 | BSI7 | VISmean | 0.186 | 0.070 | 0.008 | 1.205 | (1.051 to 1.381) | 0.536 | -0.241 |
| 1 | BSI8 | Tmean | 0.273 | 0.083 | 0.001 | 1.314 | (1.118 to 1.545) | 0.403 | -0.444 |
| 1 | BSI8 | Pmean | -0.271 | 0.073 | <0.001 | 0.763 | (0.661 to 0.880) | 0.394 | 0.321 |
| 1 | BSI8 | AHmean | 0.240 | 0.076 | 0.002 | 1.272 | (1.097 to 1.475) | 0.437 | -0.438 |
| 1 | BSI8 | VISmean | 0.084 | 0.067 | 0.207 | 1.088 | (0.955 to 1.239) | 0.483 | -0.166 |
| 1 | BSI10 | Tmean | 0.071 | 0.066 | 0.283 | 1.073 | (0.943 to 1.221) | 0.210 | -0.353 |
| 1 | BSI10 | Pmean | -0.116 | 0.064 | 0.072 | 0.891 | (0.786 to 1.010) | 0.191 | 0.273 |
| 1 | BSI10 | AHmean | 0.047 | 0.061 | 0.441 | 1.048 | (0.930 to 1.182) | 0.219 | -0.363 |
| 1 | BSI10 | VISmean | 0.114 | 0.051 | 0.026 | 1.121 | (1.014 to 1.240) | 0.213 | -0.192 |
| 2 | postive_rate | Tmean | -0.045 | 0.041 | 0.275 | 0.956 | (0.883 to 1.036) | 0.739 | -0.131 |
| 2 | postive_rate | Pmean | 0.040 | 0.038 | 0.294 | 1.041 | (0.966 to 1.121) | 0.745 | -0.005 |
| 2 | postive_rate | AHmean | -0.082 | 0.037 | 0.028 | 0.921 | (0.856 to 0.991) | 0.738 | -0.092 |
| 2 | postive_rate | VISmean | 0.057 | 0.029 | 0.048 | 1.058 | (1.001 to 1.119) | 0.745 | 0.017 |
| 2 | N_gene_positive_rate | Tmean | -0.101 | 0.075 | 0.177 | 0.904 | (0.780 to 1.047) | 1.023 | -0.061 |
| 2 | N_gene_positive_rate | Pmean | 0.013 | 0.067 | 0.849 | 1.013 | (0.888 to 1.155) | 1.007 | 0.043 |
| 2 | N_gene_positive_rate | AHmean | -0.211 | 0.070 | 0.003 | 0.81 | (0.707 to 0.929) | 1.026 | 0.098 |
| 2 | N_gene_positive_rate | VISmean | -0.005 | 0.050 | 0.916 | 0.995 | (0.902 to 1.097) | 1.003 | -0.055 |
| 2 | N_gene_concentration | Tmean | -0.104 | 0.056 | 0.065 | 0.901 | (0.807 to 1.006) | 0.701 | -0.097 |
| 2 | N_gene_concentration | Pmean | 0.151 | 0.048 | 0.002 | 1.163 | (1.058 to 1.278) | 0.740 | -0.051 |
| 2 | N_gene_concentration | AHmean | -0.159 | 0.049 | 0.001 | 0.853 | (0.775 to 0.939) | 0.702 | -0.047 |
| 2 | N_gene_concentration | VISmean | 0.021 | 0.037 | 0.571 | 1.021 | (0.950 to 1.098) | 0.676 | 0.002 |
| 2 | ORF1ab_gene_positive_rate | Tmean | -0.194 | 0.047 | <0.001 | 0.824 | (0.751 to 0.904) | 0.840 | 0.103 |
| 2 | ORF1ab_gene_positive_rate | Pmean | 0.078 | 0.038 | 0.040 | 1.082 | (1.004 to 1.166) | 0.802 | -0.139 |
| 2 | ORF1ab_gene_positive_rate | AHmean | -0.211 | 0.041 | <0.001 | 0.810 | (0.747 to 0.878) | 0.838 | 0.222 |
| 2 | ORF1ab_gene_positive_rate | VISmean | 0.004 | 0.029 | 0.887 | 1.004 | (0.949 to 1.062) | 0.761 | 0.015 |
| 2 | ORF1ab_gene_concentration | Tmean | -0.137 | 0.052 | 0.009 | 0.872 | (0.787 to 0.965) | 0.646 | 0.017 |
| 2 | ORF1ab_gene_concentration | Pmean | 0.142 | 0.043 | 0.001 | 1.153 | (1.060 to 1.254) | 0.666 | -0.086 |
| 2 | ORF1ab_gene_concentration | AHmean | -0.184 | 0.044 | <0.001 | 0.832 | (0.764 to 0.906) | 0.654 | 0.012 |
| 2 | ORF1ab_gene_concentration | VISmean | 0.065 | 0.032 | 0.046 | 1.067 | (1.001 to 1.137) | 0.615 | 0.049 |
| 2 | BSI2 | Tmean | 0.178 | 0.095 | 0.062 | 1.195 | (0.991 to 1.440) | 0.537 | -0.393 |
| 2 | BSI2 | Pmean | -0.250 | 0.095 | 0.009 | 0.779 | (0.647 to 0.938) | 0.502 | 0.233 |
| 2 | BSI2 | AHmean | 0.243 | 0.086 | 0.005 | 1.275 | (1.078 to 1.509) | 0.569 | -0.371 |
| 2 | BSI2 | VISmean | -0.047 | 0.077 | 0.54 | 0.954 | (0.821 to 1.109) | 0.527 | -0.108 |
| 2 | BSI4 | Tmean | 0.055 | 0.064 | 0.396 | 1.056 | (0.931 to 1.198) | -0.069 | -0.352 |
| 2 | BSI4 | Pmean | -0.069 | 0.061 | 0.262 | 0.934 | (0.829 to 1.052) | -0.081 | 0.252 |
| 2 | BSI4 | AHmean | 0.092 | 0.061 | 0.133 | 1.097 | (0.973 to 1.237) | -0.084 | -0.380 |
| 2 | BSI4 | VISmean | -0.023 | 0.056 | 0.682 | 0.977 | (0.876 to 1.091) | -0.045 | -0.158 |
| 2 | BSI5 | Tmean | -0.109 | 0.061 | 0.076 | 0.897 | (0.795 to 1.011) | 0.071 | -0.336 |
| 2 | BSI5 | Pmean | 0.192 | 0.059 | 0.001 | 1.212 | (1.080 to 1.360) | 0.083 | 0.235 |
| 2 | BSI5 | AHmean | -0.201 | 0.057 | <0.001 | 0.818 | (0.732 to 0.915) | 0.056 | -0.308 |
| 2 | BSI5 | VISmean | 0.122 | 0.052 | 0.018 | 1.130 | (1.021 to 1.251) | 0.021 | -0.192 |
| 2 | BSI6 | Tmean | 0.384 | 0.044 | <0.001 | 1.468 | (1.348 to 1.599) | 0.360 | -0.312 |
| 2 | BSI6 | Pmean | -0.307 | 0.032 | <0.001 | 0.736 | (0.692 to 0.783) | 0.448 | 0.130 |
| 2 | BSI6 | AHmean | 0.312 | 0.036 | <0.001 | 1.366 | (1.274 to 1.466) | 0.411 | -0.369 |
| 2 | BSI6 | VISmean | 0.030 | 0.026 | 0.245 | 1.030 | (0.980 to 1.084) | 0.402 | -0.109 |
| 2 | BSI7 | Tmean | 0.205 | 0.072 | 0.005 | 1.228 | (1.066 to 1.414) | 0.340 | -0.369 |
| 2 | BSI7 | Pmean | -0.145 | 0.072 | 0.044 | 0.865 | (0.752 to 0.996) | 0.336 | 0.222 |
| 2 | BSI7 | AHmean | 0.324 | 0.069 | <0.001 | 1.383 | (1.209 to 1.581) | 0.340 | -0.462 |
| 2 | BSI7 | VISmean | -0.006 | 0.057 | 0.917 | 0.994 | (0.890 to 1.111) | 0.367 | -0.126 |
| 2 | BSI8 | Tmean | 0.290 | 0.080 | <0.001 | 1.336 | (1.142 to 1.564) | 0.319 | -0.426 |
| 2 | BSI8 | Pmean | -0.259 | 0.077 | <0.001 | 0.772 | (0.664 to 0.897) | 0.316 | 0.276 |
| 2 | BSI8 | AHmean | 0.260 | 0.078 | <0.001 | 1.297 | (1.113 to 1.511) | 0.344 | -0.432 |
| 2 | BSI8 | VISmean | 0.132 | 0.063 | 0.036 | 1.142 | (1.009 to 1.292) | 0.407 | -0.181 |
| 2 | BSI10 | Tmean | 0.052 | 0.066 | 0.436 | 1.053 | (0.925 to 1.199) | 0.218 | -0.385 |
| 2 | BSI10 | Pmean | -0.100 | 0.061 | 0.099 | 0.905 | (0.803 to 1.019) | 0.188 | 0.258 |
| 2 | BSI10 | AHmean | 0.088 | 0.060 | 0.145 | 1.092 | (0.970 to 1.228) | 0.205 | -0.397 |
| 2 | BSI10 | VISmean | 0.065 | 0.050 | 0.195 | 1.067 | (0.968 to 1.176) | 0.214 | -0.190 |
| 3 | postive_rate | Tmean | -0.019 | 0.041 | 0.654 | 0.982 | (0.905 to 1.065) | 0.715 | -0.104 |
| 3 | postive_rate | Pmean | 0.016 | 0.038 | 0.671 | 1.016 | (0.944 to 1.095) | 0.718 | 0.057 |
| 3 | postive_rate | AHmean | -0.075 | 0.037 | 0.043 | 0.928 | (0.863 to 0.998) | 0.721 | -0.129 |
| 3 | postive_rate | VISmean | 0.024 | 0.030 | 0.416 | 1.025 | (0.967 to 1.086) | 0.726 | 0.041 |
| 3 | N_gene_positive_rate | Tmean | -0.126 | 0.077 | 0.102 | 0.882 | (0.758 to 1.025) | 1.009 | -0.072 |
| 3 | N_gene_positive_rate | Pmean | 0.008 | 0.069 | 0.908 | 1.008 | (0.881 to 1.154) | 0.986 | 0.098 |
| 3 | N_gene_positive_rate | AHmean | -0.221 | 0.071 | 0.002 | 0.801 | (0.697 to 0.921) | 1.004 | 0.025 |
| 3 | N_gene_positive_rate | VISmean | -0.027 | 0.052 | 0.603 | 0.973 | (0.878 to 1.078) | 0.988 | -0.003 |
| 3 | N_gene_concentration | Tmean | -0.128 | 0.060 | 0.033 | 0.880 | (0.783 to 0.989) | 0.666 | -0.115 |
| 3 | N_gene_concentration | Pmean | 0.166 | 0.053 | 0.002 | 1.181 | (1.064 to 1.310) | 0.702 | -0.015 |
| 3 | N_gene_concentration | AHmean | -0.180 | 0.052 | <0.001 | 0.836 | (0.755 to 0.925) | 0.669 | -0.106 |
| 3 | N_gene_concentration | VISmean | 0.017 | 0.040 | 0.668 | 1.017 | (0.941 to 1.099) | 0.638 | 0.029 |
| 3 | ORF1ab_gene_positive_rate | Tmean | -0.192 | 0.052 | <0.001 | 0.825 | (0.746 to 0.913) | 0.803 | 0.047 |
| 3 | ORF1ab_gene_positive_rate | Pmean | 0.103 | 0.043 | 0.016 | 1.108 | (1.019 to 1.205) | 0.773 | -0.092 |
| 3 | ORF1ab_gene_positive_rate | AHmean | -0.216 | 0.045 | <0.001 | 0.806 | (0.737 to 0.881) | 0.802 | 0.149 |
| 3 | ORF1ab_gene_positive_rate | VISmean | -0.007 | 0.031 | 0.814 | 0.993 | (0.934 to 1.056) | 0.729 | 0.039 |
| 3 | ORF1ab_gene_concentration | Tmean | -0.174 | 0.055 | 0.002 | 0.840 | (0.754 to 0.936) | 0.634 | -0.054 |
| 3 | ORF1ab_gene_concentration | Pmean | 0.181 | 0.048 | <0.001 | 1.199 | (1.090 to 1.318) | 0.657 | -0.059 |
| 3 | ORF1ab_gene_concentration | AHmean | -0.217 | 0.046 | <0.001 | 0.805 | (0.735 to 0.881) | 0.648 | -0.054 |
| 3 | ORF1ab_gene_concentration | VISmean | 0.048 | 0.034 | 0.16 | 1.05 | (0.981 to 1.123) | 0.581 | 0.043 |
| 3 | BSI2 | Tmean | 0.136 | 0.096 | 0.156 | 1.145 | (0.950 to 1.381) | 0.480 | -0.392 |
| 3 | BSI2 | Pmean | -0.231 | 0.091 | 0.012 | 0.794 | (0.664 to 0.950) | 0.455 | 0.251 |
| 3 | BSI2 | AHmean | 0.192 | 0.086 | 0.026 | 1.212 | (1.024 to 1.434) | 0.488 | -0.353 |
| 3 | BSI2 | VISmean | 0.053 | 0.082 | 0.519 | 1.054 | (0.898 to 1.237) | 0.461 | -0.118 |
| 3 | BSI4 | Tmean | 0.047 | 0.063 | 0.453 | 1.049 | (0.927 to 1.187) | -0.043 | -0.344 |
| 3 | BSI4 | Pmean | -0.023 | 0.064 | 0.726 | 0.978 | (0.862 to 1.109) | -0.049 | 0.236 |
| 3 | BSI4 | AHmean | 0.055 | 0.061 | 0.371 | 1.056 | (0.937 to 1.191) | -0.041 | -0.357 |
| 3 | BSI4 | VISmean | 0.028 | 0.051 | 0.585 | 1.028 | (0.931 to 1.136) | -0.04 | -0.167 |
| 3 | BSI5 | Tmean | -0.051 | 0.063 | 0.421 | 0.950 | (0.840 to 1.076) | 0.045 | -0.323 |
| 3 | BSI5 | Pmean | 0.141 | 0.061 | 0.022 | 1.151 | (1.021 to 1.298) | 0.063 | 0.184 |
| 3 | BSI5 | AHmean | -0.102 | 0.056 | 0.071 | 0.903 | (0.809 to 1.009) | 0.038 | -0.324 |
| 3 | BSI5 | VISmean | 0.035 | 0.052 | 0.505 | 1.036 | (0.934 to 1.148) | 0.034 | -0.174 |
| 3 | BSI6 | Tmean | 0.358 | 0.050 | <0.001 | 1.431 | (1.296 to 1.579) | 0.316 | -0.351 |
| 3 | BSI6 | Pmean | -0.289 | 0.04 | <0.001 | 0.749 | (0.693 to 0.810) | 0.409 | 0.051 |
| 3 | BSI6 | AHmean | 0.275 | 0.037 | <0.001 | 1.317 | (1.225 to 1.415) | 0.417 | -0.289 |
| 3 | BSI6 | VISmean | -0.001 | 0.026 | 0.979 | 0.999 | (0.951 to 1.051) | 0.401 | -0.137 |
| 3 | BSI7 | Tmean | 0.270 | 0.075 | <0.001 | 1.310 | (1.132 to 1.517) | 0.338 | -0.426 |
| 3 | BSI7 | Pmean | -0.126 | 0.08 | 0.114 | 0.882 | (0.754 to 1.031) | 0.359 | 0.217 |
| 3 | BSI7 | AHmean | 0.290 | 0.072 | <0.001 | 1.336 | (1.160 to 1.539) | 0.362 | -0.429 |
| 3 | BSI7 | VISmean | 0.108 | 0.057 | 0.060 | 1.114 | (0.996 to 1.247) | 0.401 | -0.195 |
| 3 | BSI8 | Tmean | 0.220 | 0.079 | 0.006 | 1.246 | (1.067 to 1.454) | 0.294 | -0.389 |
| 3 | BSI8 | Pmean | -0.152 | 0.077 | 0.048 | 0.859 | (0.739 to 0.998) | 0.296 | 0.215 |
| 3 | BSI8 | AHmean | 0.219 | 0.076 | 0.004 | 1.245 | (1.073 to 1.445) | 0.297 | -0.402 |
| 3 | BSI8 | VISmean | 0.053 | 0.056 | 0.348 | 1.054 | (0.944 to 1.177) | 0.360 | -0.151 |
| 3 | BSI10 | Tmean | 0.097 | 0.066 | 0.145 | 1.102 | (0.968 to 1.255) | 0.205 | -0.418 |
| 3 | BSI10 | Pmean | -0.091 | 0.061 | 0.140 | 0.913 | (0.810 to 1.030) | 0.199 | 0.281 |
| 3 | BSI10 | AHmean | 0.128 | 0.06 | 0.032 | 1.136 | (1.011 to 1.277) | 0.200 | -0.437 |
| 3 | BSI10 | VISmean | -0.053 | 0.052 | 0.310 | 0.948 | (0.856 to 1.051) | 0.225 | -0.145 |
| 4 | postive_rate | Tmean | -0.046 | 0.046 | 0.312 | 0.955 | (0.873 to 1.044) | 0.685 | -0.108 |
| 4 | postive_rate | Pmean | 0.034 | 0.042 | 0.431 | 1.034 | (0.952 to 1.124) | 0.687 | 0.060 |
| 4 | postive_rate | AHmean | -0.116 | 0.041 | 0.005 | 0.891 | (0.823 to 0.965) | 0.693 | -0.118 |
| 4 | postive_rate | VISmean | 0.019 | 0.031 | 0.546 | 1.019 | (0.959 to 1.082) | 0.693 | 0.062 |
| 4 | N_gene_positive_rate | Tmean | -0.133 | 0.078 | 0.089 | 0.876 | (0.752 to 1.020) | 0.983 | -0.098 |
| 4 | N_gene_positive_rate | Pmean | -0.001 | 0.070 | 0.991 | 0.999 | (0.871 to 1.146) | 0.958 | 0.153 |
| 4 | N_gene_positive_rate | AHmean | -0.197 | 0.071 | 0.006 | 0.821 | (0.714 to 0.944) | 0.971 | -0.069 |
| 4 | N_gene_positive_rate | VISmean | -0.054 | 0.053 | 0.313 | 0.948 | (0.854 to 1.052) | 0.965 | 0.033 |
| 4 | N_gene_concentration | Tmean | -0.121 | 0.062 | 0.053 | 0.886 | (0.784 to 1.001) | 0.625 | -0.162 |
| 4 | N_gene_concentration | Pmean | 0.153 | 0.057 | 0.007 | 1.166 | (1.042 to 1.303) | 0.655 | 0.046 |
| 4 | N_gene_concentration | AHmean | -0.163 | 0.054 | 0.003 | 0.850 | (0.764 to 0.945) | 0.630 | -0.195 |
| 4 | N_gene_concentration | VISmean | 0.008 | 0.041 | 0.848 | 1.008 | (0.929 to 1.093) | 0.602 | 0.046 |
| 4 | ORF1ab_gene_positive_rate | Tmean | -0.169 | 0.056 | 0.002 | 0.844 | (0.757 to 0.942) | 0.758 | -0.015 |
| 4 | ORF1ab_gene_positive_rate | Pmean | 0.109 | 0.048 | 0.023 | 1.115 | (1.015 to 1.225) | 0.740 | -0.037 |
| 4 | ORF1ab_gene_positive_rate | AHmean | -0.191 | 0.049 | <0.001 | 0.826 | (0.750 to 0.910) | 0.757 | 0.049 |
| 4 | ORF1ab_gene_positive_rate | VISmean | -0.024 | 0.033 | 0.475 | 0.977 | (0.915 to 1.042) | 0.698 | 0.057 |
| 4 | ORF1ab_gene_concentration | Tmean | -0.189 | 0.058 | 0.001 | 0.828 | (0.739 to 0.928) | 0.615 | -0.120 |
| 4 | ORF1ab_gene_concentration | Pmean | 0.197 | 0.054 | <0.001 | 1.217 | (1.096 to 1.353) | 0.634 | -0.018 |
| 4 | ORF1ab_gene_concentration | AHmean | -0.225 | 0.049 | <0.001 | 0.799 | (0.726 to 0.879) | 0.632 | -0.126 |
| 4 | ORF1ab_gene_concentration | VISmean | 0.026 | 0.036 | 0.472 | 1.026 | (0.956 to 1.102) | 0.544 | 0.035 |
| 4 | BSI2 | Tmean | 0.075 | 0.095 | 0.435 | 1.077 | (0.894 to 1.299) | 0.384 | -0.395 |
| 4 | BSI2 | Pmean | -0.036 | 0.087 | 0.678 | 0.965 | (0.813 to 1.144) | 0.385 | 0.278 |
| 4 | BSI2 | AHmean | 0.144 | 0.085 | 0.093 | 1.155 | (0.977 to 1.365) | 0.396 | -0.376 |
| 4 | BSI2 | VISmean | -0.077 | 0.084 | 0.360 | 0.926 | (0.786 to 1.092) | 0.355 | -0.136 |
| 4 | BSI4 | Tmean | 0.032 | 0.065 | 0.622 | 1.032 | (0.909 to 1.172) | -0.022 | -0.341 |
| 4 | BSI4 | Pmean | -0.018 | 0.064 | 0.781 | 0.983 | (0.868 to 1.113) | -0.025 | 0.234 |
| 4 | BSI4 | AHmean | 0.065 | 0.061 | 0.285 | 1.067 | (0.947 to 1.202) | -0.021 | -0.365 |
| 4 | BSI4 | VISmean | 0.013 | 0.051 | 0.800 | 1.013 | (0.917 to 1.118) | -0.031 | -0.165 |
| 4 | BSI5 | Tmean | -0.059 | 0.065 | 0.365 | 0.943 | (0.831 to 1.070) | 0.058 | -0.324 |
| 4 | BSI5 | Pmean | 0.189 | 0.064 | 0.003 | 1.208 | (1.067 to 1.368) | 0.099 | 0.165 |
| 4 | BSI5 | AHmean | -0.070 | 0.058 | 0.233 | 0.933 | (0.832 to 1.046) | 0.057 | -0.330 |
| 4 | BSI5 | VISmean | -0.095 | 0.051 | 0.062 | 0.909 | (0.823 to 1.004) | 0.047 | -0.135 |
| 4 | BSI6 | Tmean | 0.373 | 0.052 | <0.001 | 1.452 | (1.311 to 1.609) | 0.321 | -0.312 |
| 4 | BSI6 | Pmean | -0.228 | 0.043 | <0.001 | 0.797 | (0.733 to 0.866) | 0.378 | 0.063 |
| 4 | BSI6 | AHmean | 0.286 | 0.043 | <0.001 | 1.330 | (1.223 to 1.447) | 0.376 | -0.357 |
| 4 | BSI6 | VISmean | 0.089 | 0.029 | 0.002 | 1.093 | (1.032 to 1.158) | 0.412 | -0.102 |
| 4 | BSI7 | Tmean | 0.277 | 0.078 | <0.001 | 1.319 | (1.132 to 1.537) | 0.277 | -0.410 |
| 4 | BSI7 | Pmean | -0.143 | 0.081 | 0.077 | 0.867 | (0.740 to 1.015) | 0.310 | 0.228 |
| 4 | BSI7 | AHmean | 0.250 | 0.074 | <0.001 | 1.284 | (1.111 to 1.483) | 0.316 | -0.427 |
| 4 | BSI7 | VISmean | 0.091 | 0.062 | 0.144 | 1.095 | (0.970 to 1.237) | 0.333 | -0.139 |
| 4 | BSI8 | Tmean | 0.214 | 0.078 | 0.007 | 1.238 | (1.062 to 1.444) | 0.320 | -0.351 |
| 4 | BSI8 | Pmean | -0.091 | 0.076 | 0.233 | 0.913 | (0.786 to 1.060) | 0.357 | 0.192 |
| 4 | BSI8 | AHmean | 0.196 | 0.075 | 0.009 | 1.217 | (1.051 to 1.409) | 0.335 | -0.371 |
| 4 | BSI8 | VISmean | 0.011 | 0.062 | 0.854 | 1.012 | (0.896 to 1.142) | 0.389 | -0.122 |
| 4 | BSI10 | Tmean | 0.186 | 0.067 | 0.006 | 1.205 | (1.057 to 1.373) | 0.181 | -0.426 |
| 4 | BSI10 | Pmean | -0.151 | 0.062 | 0.016 | 0.860 | (0.761 to 0.972) | 0.194 | 0.299 |
| 4 | BSI10 | AHmean | 0.192 | 0.061 | 0.002 | 1.212 | (1.075 to 1.366) | 0.204 | -0.465 |
| 4 | BSI10 | VISmean | -0.096 | 0.051 | 0.059 | 0.909 | (0.823 to 1.003) | 0.225 | -0.100 |
| 5 | postive_rate | Tmean | -0.102 | 0.050 | 0.042 | 0.903 | (0.820 to 0.996) | 0.652 | -0.151 |
| 5 | postive_rate | Pmean | 0.096 | 0.047 | 0.041 | 1.101 | (1.004 to 1.207) | 0.661 | 0.039 |
| 5 | postive_rate | AHmean | -0.158 | 0.043 | <0.001 | 0.854 | (0.784 to 0.929) | 0.653 | -0.116 |
| 5 | postive_rate | VISmean | 0.026 | 0.034 | 0.452 | 1.026 | (0.960 to 1.097) | 0.641 | 0.039 |
| 5 | N_gene_positive_rate | Tmean | -0.129 | 0.079 | 0.106 | 0.879 | (0.753 to 1.027) | 0.975 | -0.130 |
| 5 | N_gene_positive_rate | Pmean | 0.004 | 0.071 | 0.961 | 1.004 | (0.873 to 1.154) | 0.952 | 0.179 |
| 5 | N_gene_positive_rate | AHmean | -0.176 | 0.072 | 0.015 | 0.839 | (0.728 to 0.966) | 0.958 | -0.139 |
| 5 | N_gene_positive_rate | VISmean | -0.059 | 0.053 | 0.265 | 0.943 | (0.850 to 1.046) | 0.953 | 0.024 |
| 5 | N_gene_concentration | Tmean | -0.073 | 0.065 | 0.259 | 0.930 | (0.819 to 1.055) | 0.581 | -0.216 |
| 5 | N_gene_concentration | Pmean | 0.104 | 0.060 | 0.083 | 1.110 | (0.987 to 1.249) | 0.604 | 0.108 |
| 5 | N_gene_concentration | AHmean | -0.102 | 0.055 | 0.065 | 0.903 | (0.811 to 1.006) | 0.587 | -0.278 |
| 5 | N_gene_concentration | VISmean | -0.014 | 0.042 | 0.741 | 0.986 | (0.909 to 1.070) | 0.573 | 0.051 |
| 5 | ORF1ab_gene_positive_rate | Tmean | -0.145 | 0.059 | 0.015 | 0.865 | (0.771 to 0.972) | 0.721 | -0.052 |
| 5 | ORF1ab_gene_positive_rate | Pmean | 0.101 | 0.053 | 0.056 | 1.106 | (0.998 to 1.227) | 0.710 | 0.008 |
| 5 | ORF1ab_gene_positive_rate | AHmean | -0.162 | 0.052 | 0.002 | 0.850 | (0.768 to 0.942) | 0.719 | -0.040 |
| 5 | ORF1ab_gene_positive_rate | VISmean | -0.030 | 0.035 | 0.390 | 0.970 | (0.906 to 1.039) | 0.672 | 0.065 |
| 5 | ORF1ab_gene_concentration | Tmean | -0.142 | 0.06 | 0.018 | 0.868 | (0.772 to 0.976) | 0.569 | -0.168 |
| 5 | ORF1ab_gene_concentration | Pmean | 0.148 | 0.057 | 0.010 | 1.160 | (1.037 to 1.297) | 0.582 | 0.034 |
| 5 | ORF1ab_gene_concentration | AHmean | -0.167 | 0.050 | <0.001 | 0.846 | (0.767 to 0.933) | 0.583 | -0.196 |
| 5 | ORF1ab_gene_concentration | VISmean | -0.007 | 0.037 | 0.847 | 0.993 | (0.924 to 1.067) | 0.515 | 0.030 |
| 5 | BSI2 | Tmean | 0.158 | 0.097 | 0.105 | 1.171 | (0.968 to 1.417) | 0.250 | -0.342 |
| 5 | BSI2 | Pmean | -0.056 | 0.092 | 0.545 | 0.946 | (0.79 to 1.132) | 0.260 | 0.233 |
| 5 | BSI2 | AHmean | 0.072 | 0.089 | 0.415 | 1.075 | (0.904 to 1.279) | 0.283 | -0.359 |
| 5 | BSI2 | VISmean | 0.234 | 0.079 | 0.003 | 1.263 | (1.082 to 1.474) | 0.238 | -0.067 |
| 5 | BSI4 | Tmean | -0.032 | 0.065 | 0.627 | 0.969 | (0.853 to 1.101) | -0.014 | -0.339 |
| 5 | BSI4 | Pmean | 0.000 | 0.064 | 0.995 | 1.000 | (0.882 to 1.134) | -0.019 | 0.231 |
| 5 | BSI4 | AHmean | 0.049 | 0.06 | 0.410 | 1.050 | (0.935 to 1.181) | -0.025 | -0.355 |
| 5 | BSI4 | VISmean | -0.028 | 0.051 | 0.582 | 0.972 | (0.879 to 1.075) | -0.038 | -0.170 |
| 5 | BSI5 | Tmean | 0.008 | 0.064 | 0.898 | 1.008 | (0.89 to 1.142) | 0.007 | -0.343 |
| 5 | BSI5 | Pmean | 0.127 | 0.061 | 0.038 | 1.135 | (1.008 to 1.278) | 0.039 | 0.206 |
| 5 | BSI5 | AHmean | 0.029 | 0.057 | 0.614 | 1.029 | (0.920 to 1.152) | 0.005 | -0.360 |
| 5 | BSI5 | VISmean | -0.106 | 0.050 | 0.033 | 0.899 | (0.816 to 0.991) | 0.010 | -0.142 |
| 5 | BSI6 | Tmean | 0.401 | 0.054 | <0.001 | 1.494 | (1.343 to 1.662) | 0.284 | -0.359 |
| 5 | BSI6 | Pmean | -0.275 | 0.039 | <0.001 | 0.759 | (0.703 to 0.820) | 0.369 | 0.117 |
| 5 | BSI6 | AHmean | 0.336 | 0.045 | <0.001 | 1.400 | (1.281 to 1.529) | 0.356 | -0.437 |
| 5 | BSI6 | VISmean | 0.092 | 0.032 | 0.004 | 1.096 | (1.030 to 1.167) | 0.394 | -0.073 |
| 5 | BSI7 | Tmean | 0.309 | 0.073 | <0.001 | 1.362 | (1.182 to 1.570) | 0.311 | -0.382 |
| 5 | BSI7 | Pmean | -0.276 | 0.075 | <0.001 | 0.759 | (0.655 to 0.879) | 0.330 | 0.291 |
| 5 | BSI7 | AHmean | 0.348 | 0.073 | <0.001 | 1.416 | (1.227 to 1.635) | 0.346 | -0.502 |
| 5 | BSI7 | VISmean | 0.086 | 0.067 | 0.197 | 1.090 | (0.956 to 1.243) | 0.372 | -0.113 |
| 5 | BSI8 | Tmean | 0.206 | 0.079 | 0.009 | 1.228 | (1.053 to 1.433) | 0.258 | -0.356 |
| 5 | BSI8 | Pmean | -0.062 | 0.082 | 0.450 | 0.940 | (0.801 to 1.103) | 0.303 | 0.185 |
| 5 | BSI8 | AHmean | 0.202 | 0.078 | 0.01 | 1.223 | (1.051 to 1.424) | 0.277 | -0.402 |
| 5 | BSI8 | VISmean | -0.041 | 0.054 | 0.441 | 0.959 | (0.864 to 1.066) | 0.324 | -0.078 |
| 5 | BSI10 | Tmean | 0.225 | 0.066 | <0.001 | 1.252 | (1.101 to 1.424) | 0.126 | -0.489 |
| 5 | BSI10 | Pmean | -0.187 | 0.063 | 0.003 | 0.830 | (0.734 to 0.938) | 0.118 | 0.313 |
| 5 | BSI10 | AHmean | 0.237 | 0.062 | <0.001 | 1.268 | (1.122 to 1.432) | 0.142 | -0.495 |
| 5 | BSI10 | VISmean | -0.121 | 0.054 | 0.024 | 0.886 | (0.797 to 0.984) | 0.174 | -0.111 |
| 6 | postive_rate | Tmean | -0.156 | 0.054 | 0.004 | 0.855 | (0.770 to 0.950) | 0.646 | -0.157 |
| 6 | postive_rate | Pmean | 0.174 | 0.051 | <0.001 | 1.190 | (1.077 to 1.315) | 0.672 | 0.023 |
| 6 | postive_rate | AHmean | -0.196 | 0.045 | <0.001 | 0.822 | (0.753 to 0.898) | 0.643 | -0.123 |
| 6 | postive_rate | VISmean | 0.015 | 0.036 | 0.683 | 1.015 | (0.945 to 1.090) | 0.614 | 0.029 |
| 6 | N_gene_positive_rate | Tmean | -0.108 | 0.081 | 0.181 | 0.897 | (0.766 to 1.051) | 0.948 | -0.161 |
| 6 | N_gene_positive_rate | Pmean | 0.008 | 0.075 | 0.919 | 1.008 | (0.870 to 1.167) | 0.932 | 0.194 |
| 6 | N_gene_positive_rate | AHmean | -0.165 | 0.073 | 0.025 | 0.848 | (0.734 to 0.979) | 0.935 | -0.173 |
| 6 | N_gene_positive_rate | VISmean | -0.042 | 0.053 | 0.431 | 0.959 | (0.864 to 1.065) | 0.925 | -0.012 |
| 6 | N_gene_concentration | Tmean | -0.014 | 0.067 | 0.832 | 0.986 | (0.866 to 1.123) | 0.531 | -0.249 |
| 6 | N_gene_concentration | Pmean | 0.027 | 0.063 | 0.676 | 1.027 | (0.907 to 1.163) | 0.539 | 0.165 |
| 6 | N_gene_concentration | AHmean | -0.042 | 0.057 | 0.462 | 0.959 | (0.858 to 1.072) | 0.540 | -0.327 |
| 6 | N_gene_concentration | VISmean | -0.029 | 0.042 | 0.490 | 0.971 | (0.895 to 1.055) | 0.538 | 0.044 |
| 6 | ORF1ab_gene_positive_rate | Tmean | -0.123 | 0.063 | 0.051 | 0.884 | (0.782 to 1.000) | 0.680 | -0.069 |
| 6 | ORF1ab_gene_positive_rate | Pmean | 0.073 | 0.058 | 0.211 | 1.075 | (0.960 to 1.205) | 0.664 | 0.044 |
| 6 | ORF1ab_gene_positive_rate | AHmean | -0.130 | 0.055 | 0.018 | 0.878 | (0.788 to 0.978) | 0.677 | -0.106 |
| 6 | ORF1ab_gene_positive_rate | VISmean | -0.045 | 0.036 | 0.214 | 0.956 | (0.891 to 1.026) | 0.639 | 0.068 |
| 6 | ORF1ab_gene_concentration | Tmean | -0.080 | 0.061 | 0.191 | 0.923 | (0.819 to 1.041) | 0.512 | -0.191 |
| 6 | ORF1ab_gene_concentration | Pmean | 0.063 | 0.060 | 0.292 | 1.065 | (0.947 to 1.198) | 0.509 | 0.090 |
| 6 | ORF1ab_gene_concentration | AHmean | -0.092 | 0.051 | 0.074 | 0.912 | (0.825 to 1.009) | 0.518 | -0.247 |
| 6 | ORF1ab_gene_concentration | VISmean | -0.028 | 0.037 | 0.453 | 0.973 | (0.905 to 1.045) | 0.486 | 0.027 |
| 6 | BSI2 | Tmean | 0.154 | 0.096 | 0.109 | 1.166 | (0.967 to 1.407) | 0.192 | -0.326 |
| 6 | BSI2 | Pmean | -0.073 | 0.090 | 0.421 | 0.930 | (0.780 to 1.110) | 0.201 | 0.224 |
| 6 | BSI2 | AHmean | 0.112 | 0.089 | 0.207 | 1.118 | (0.940 to 1.330) | 0.236 | -0.346 |
| 6 | BSI2 | VISmean | 0.157 | 0.081 | 0.053 | 1.169 | (0.999 to 1.369) | 0.171 | -0.098 |
| 6 | BSI4 | Tmean | 0.023 | 0.065 | 0.725 | 1.023 | (0.901 to 1.162) | 0.008 | -0.346 |
| 6 | BSI4 | Pmean | -0.027 | 0.065 | 0.683 | 0.974 | (0.857 to 1.107) | 0.001 | 0.235 |
| 6 | BSI4 | AHmean | 0.089 | 0.058 | 0.129 | 1.093 | (0.975 to 1.225) | 0.003 | -0.375 |
| 6 | BSI4 | VISmean | -0.072 | 0.054 | 0.179 | 0.930 | (0.837 to 1.034) | -0.014 | -0.174 |
| 6 | BSI5 | Tmean | 0.030 | 0.066 | 0.648 | 1.031 | (0.906 to 1.173) | -0.007 | -0.349 |
| 6 | BSI5 | Pmean | 0.097 | 0.061 | 0.116 | 1.101 | (0.977 to 1.242) | 0.030 | 0.223 |
| 6 | BSI5 | AHmean | 0.027 | 0.058 | 0.646 | 1.027 | (0.916 to 1.151) | 0.012 | -0.360 |
| 6 | BSI5 | VISmean | -0.153 | 0.048 | 0.002 | 0.858 | (0.781 to 0.943) | -0.006 | -0.144 |
| 6 | BSI6 | Tmean | 0.349 | 0.053 | <0.001 | 1.418 | (1.279 to 1.572) | 0.311 | -0.298 |
| 6 | BSI6 | Pmean | -0.288 | 0.039 | <0.001 | 0.750 | (0.695 to 0.809) | 0.380 | 0.146 |
| 6 | BSI6 | AHmean | 0.351 | 0.044 | <0.001 | 1.420 | (1.304 to 1.548) | 0.383 | -0.421 |
| 6 | BSI6 | VISmean | 0.049 | 0.041 | 0.227 | 1.051 | (0.970 to 1.138) | 0.405 | -0.016 |
| 6 | BSI7 | Tmean | 0.280 | 0.067 | <0.001 | 1.323 | (1.160 to 1.508) | 0.329 | -0.347 |
| 6 | BSI7 | Pmean | -0.317 | 0.072 | <0.001 | 0.728 | (0.632 to 0.838) | 0.333 | 0.318 |
| 6 | BSI7 | AHmean | 0.387 | 0.073 | <0.001 | 1.472 | (1.276 to 1.698) | 0.359 | -0.491 |
| 6 | BSI7 | VISmean | 0.055 | 0.076 | 0.463 | 1.057 | (0.912 to 1.226) | 0.374 | -0.13 |
| 6 | BSI8 | Tmean | 0.257 | 0.082 | 0.002 | 1.293 | (1.101 to 1.518) | 0.227 | -0.396 |
| 6 | BSI8 | Pmean | -0.180 | 0.082 | 0.030 | 0.836 | (0.711 to 0.982) | 0.234 | 0.252 |
| 6 | BSI8 | AHmean | 0.345 | 0.081 | <0.001 | 1.412 | (1.204 to 1.656) | 0.241 | -0.48 |
| 6 | BSI8 | VISmean | -0.135 | 0.064 | 0.036 | 0.874 | (0.771 to 0.991) | 0.304 | -0.09 |
| 6 | BSI10 | Tmean | 0.200 | 0.063 | 0.002 | 1.221 | (1.079 to 1.382) | 0.108 | -0.453 |
| 6 | BSI10 | Pmean | -0.153 | 0.062 | 0.014 | 0.858 | (0.760 to 0.969) | 0.098 | 0.296 |
| 6 | BSI10 | AHmean | 0.195 | 0.062 | 0.002 | 1.215 | (1.075 to 1.373) | 0.117 | -0.461 |
| 6 | BSI10 | VISmean | -0.045 | 0.054 | 0.407 | 0.956 | (0.860 to 1.063) | 0.157 | -0.153 |
| 7 | postive_rate | Tmean | -0.182 | 0.059 | 0.002 | 0.834 | (0.743 to 0.936) | 0.633 | -0.204 |
| 7 | postive_rate | Pmean | 0.238 | 0.057 | <0.001 | 1.268 | (1.134 to 1.418) | 0.680 | 0.055 |
| 7 | postive_rate | AHmean | -0.223 | 0.049 | <0.001 | 0.800 | (0.727 to 0.881) | 0.634 | -0.17 |
| 7 | postive_rate | VISmean | 0.006 | 0.037 | 0.877 | 1.006 | (0.935 to 1.081) | 0.587 | 0.019 |
| 7 | N_gene_positive_rate | Tmean | -0.120 | 0.080 | 0.133 | 0.887 | (0.758 to 1.037) | 0.916 | -0.158 |
| 7 | N_gene_positive_rate | Pmean | 0.030 | 0.077 | 0.698 | 1.030 | (0.886 to 1.198) | 0.904 | 0.186 |
| 7 | N_gene_positive_rate | AHmean | -0.19 | 0.072 | 0.009 | 0.827 | (0.718 to 0.953) | 0.906 | -0.166 |
| 7 | N_gene_positive_rate | VISmean | -0.047 | 0.053 | 0.382 | 0.955 | (0.860 to 1.059) | 0.887 | -0.026 |
| 7 | N_gene_concentration | Tmean | 0.018 | 0.067 | 0.787 | 1.018 | (0.893 to 1.161) | 0.485 | -0.254 |
| 7 | N_gene_concentration | Pmean | -0.046 | 0.066 | 0.485 | 0.955 | (0.840 to 1.087) | 0.476 | 0.201 |
| 7 | N_gene_concentration | AHmean | -0.001 | 0.058 | 0.983 | 0.999 | (0.892 to 1.118) | 0.493 | -0.336 |
| 7 | N_gene_concentration | VISmean | -0.048 | 0.043 | 0.259 | 0.953 | (0.877 to 1.036) | 0.497 | 0.031 |
| 7 | ORF1ab_gene_positive_rate | Tmean | -0.109 | 0.066 | 0.098 | 0.897 | (0.788 to 1.020) | 0.634 | -0.088 |
| 7 | ORF1ab_gene_positive_rate | Pmean | 0.035 | 0.063 | 0.574 | 1.036 | (0.916 to 1.172) | 0.604 | 0.079 |
| 7 | ORF1ab_gene_positive_rate | AHmean | -0.103 | 0.057 | 0.073 | 0.902 | (0.806 to 1.009) | 0.628 | -0.156 |
| 7 | ORF1ab_gene_positive_rate | VISmean | -0.061 | 0.037 | 0.101 | 0.941 | (0.876 to 1.012) | 0.597 | 0.062 |
| 7 | ORF1ab_gene_concentration | Tmean | -0.029 | 0.062 | 0.641 | 0.972 | (0.862 to 1.096) | 0.456 | -0.201 |
| 7 | ORF1ab_gene_concentration | Pmean | -0.018 | 0.063 | 0.772 | 0.982 | (0.869 to 1.110) | 0.439 | 0.131 |
| 7 | ORF1ab_gene_concentration | AHmean | -0.025 | 0.052 | 0.634 | 0.976 | (0.881 to 1.081) | 0.455 | -0.271 |
| 7 | ORF1ab_gene_concentration | VISmean | -0.044 | 0.037 | 0.243 | 0.957 | (0.890 to 1.030) | 0.452 | 0.016 |
| 7 | BSI2 | Tmean | 0.110 | 0.089 | 0.215 | 1.117 | (0.938 to 1.329) | 0.159 | -0.330 |
| 7 | BSI2 | Pmean | -0.054 | 0.088 | 0.539 | 0.947 | (0.797 to 1.126) | 0.165 | 0.233 |
| 7 | BSI2 | AHmean | 0.122 | 0.088 | 0.163 | 1.130 | (0.952 to 1.342) | 0.188 | -0.337 |
| 7 | BSI2 | VISmean | 0.053 | 0.083 | 0.523 | 1.055 | (0.896 to 1.242) | 0.144 | -0.136 |
| 7 | BSI4 | Tmean | 0.005 | 0.065 | 0.934 | 1.005 | (0.886 to 1.141) | -0.004 | -0.342 |
| 7 | BSI4 | Pmean | -0.006 | 0.059 | 0.917 | 0.994 | (0.885 to 1.116) | -0.013 | 0.234 |
| 7 | BSI4 | AHmean | 0.053 | 0.059 | 0.364 | 1.055 | (0.94 to 1.184) | -0.016 | -0.369 |
| 7 | BSI4 | VISmean | 0.046 | 0.053 | 0.392 | 1.047 | (0.943 to 1.162) | -0.013 | -0.170 |
| 7 | BSI5 | Tmean | 0.032 | 0.067 | 0.636 | 1.032 | (0.906 to 1.176) | -0.028 | -0.341 |
| 7 | BSI5 | Pmean | -0.082 | 0.063 | 0.191 | 0.921 | (0.814 to 1.042) | -0.035 | 0.244 |
| 7 | BSI5 | AHmean | 0.043 | 0.059 | 0.464 | 1.044 | (0.930 to 1.172) | -0.012 | -0.350 |
| 7 | BSI5 | VISmean | -0.078 | 0.05 | 0.118 | 0.925 | (0.839 to 1.020) | -0.045 | -0.159 |
| 7 | BSI6 | Tmean | 0.282 | 0.054 | <0.001 | 1.326 | (1.192 to 1.475) | 0.309 | -0.320 |
| 7 | BSI6 | Pmean | -0.250 | 0.042 | <0.001 | 0.779 | (0.718 to 0.845) | 0.346 | 0.209 |
| 7 | BSI6 | AHmean | 0.320 | 0.045 | <0.001 | 1.377 | (1.262 to 1.504) | 0.364 | -0.441 |
| 7 | BSI6 | VISmean | -0.003 | 0.043 | 0.945 | 0.997 | (0.917 to 1.084) | 0.403 | -0.037 |
| 7 | BSI7 | Tmean | 0.228 | 0.068 | <0.001 | 1.256 | (1.099 to 1.436) | 0.361 | -0.328 |
| 7 | BSI7 | Pmean | -0.266 | 0.074 | <0.001 | 0.766 | (0.662 to 0.886) | 0.351 | 0.286 |
| 7 | BSI7 | AHmean | 0.342 | 0.076 | <0.001 | 1.408 | (1.213 to 1.634) | 0.385 | -0.422 |
| 7 | BSI7 | VISmean | 0.084 | 0.074 | 0.258 | 1.088 | (0.940 to 1.258) | 0.401 | -0.174 |
| 7 | BSI8 | Tmean | 0.208 | 0.085 | <0.001 | 1.231 | (1.043 to 1.453) | 0.301 | -0.375 |
| 7 | BSI8 | Pmean | -0.233 | 0.089 | 0.002 | 0.792 | (0.665 to 0.944) | 0.284 | 0.302 |
| 7 | BSI8 | AHmean | 0.324 | 0.084 | 0.002 | 1.382 | (1.173 to 1.630) | 0.316 | -0.483 |
| 7 | BSI8 | VISmean | -0.147 | 0.061 | 0.008 | 0.863 | (0.766 to 0.973) | 0.348 | -0.049 |
| 7 | BSI10 | Tmean | 0.104 | 0.063 | 0.711 | 1.109 | (0.981 to 1.254) | 0.167 | -0.396 |
| 7 | BSI10 | Pmean | -0.117 | 0.062 | 0.578 | 0.890 | (0.788 to 1.004) | 0.150 | 0.283 |
| 7 | BSI10 | AHmean | 0.131 | 0.061 | 0.887 | 1.139 | (1.012 to 1.283) | 0.169 | -0.424 |
| 7 | BSI10 | VISmean | -0.060 | 0.050 | 0.083 | 0.942 | (0.855 to 1.039) | 0.185 | -0.139 |
